# South Brazilian Amber Propolis: Chemical Profile, and Antioxidant, Antimicrobial and Antileukemic Activities

**DOI:** 10.3390/molecules31183143

**Published:** 2026-09-08

**Authors:** Viviane Ulbrich-Ferreira, Tanira da Silveira Prieto, Josiely Pereira Machado, Adriano Alves de Paula, Jeferson Luis Franco, Nélson Rodrigues de Carvalho, Elton Luís Gasparotto Denardin, Jefferson Soares, Juliano Tomazoni Boldo, Susiane Cavinatto Meira, Helmoz Roseniaim Appelt, Andrés Delgado-Cañedo

**Affiliations:** 1 Interdisciplinary Center for Biotechnology Research (CIPBIOTEC), Federal University of Pampa (UNIPAMPA), São Gabriel Campus, São Gabriel 97307-020, RS, Braziltaniraprieto.aluno@unipampa.edu.br (T.d.S.P.); josy-sg@hotmail.com (J.P.M.); julianoboldo@unipampa.edu.br (J.T.B.);; 2 Laboratory of Studies in Phytochemistry and Natural Products (LEFQPN), Federal University of Pampa (UNIPAMPA), Uruguaiana Campus, Uruguaiana 97501-970, RS, Brazil

**Keywords:** propolis, antimicrobial agents, antineoplasics, nutraceutics, terpenes, Pampa biome

## Abstract

This study introduces “amber propolis,” a unique variety from the Pampa biome in southern Brazil, characterized by a distinct chemical fingerprint and high biological potency. The composition was comprehensively analyzed via GC-MS and LC-MS/MS; biological activity was evaluated through antimicrobial (*Staphylococcus aureus* and *Escherichia coli*) and antileukemic screenings against K562, Jurkat, and U937 cell lines. Results revealed a terpenoid-dominant profile (comprising 60–70% of identified features) despite a lower flavonoid abundance compared to traditional reference samples. Amber propolis exhibited significantly higher antibacterial activity against *S. aureus* than green propolis, whereas all varieties showed similar dose-dependent inhibition of *E. coli*, reaching complete growth suppression at 500 µg/mL. Notably, it acted as a potent antileukemic agent, inducing rapid apoptosis starting at 12 h in Jurkat and U937 cell lines, and at 24 h for K562 cells, while concomitantly triggering G2/M cell cycle arrest. Unlike the variable responses observed for green and red propolis across lineages, amber propolis demonstrated a uniquely uniform cytotoxic effect, suggesting the involvement of distinct, conserved molecular mechanisms. These findings challenge the prevailing paradigm associating propolis bioactivity primarily with phenolic abundance. Collectively, this study establishes amber propolis as a chemically distinct and biologically active variety with strong potential for pharmaceutical and nutraceutical applications, supporting its future valorization and geographical indication within the Brazilian Pampa biome.

## 1. Introduction

Propolis or “bee glue” is a resinous substance produced by bees in order to protect the hive. It is a complex resinous substance produced from plant exudates, widely recognized for its diverse chemical composition and several biological activities [1,2,3,4,5]. The most extensively investigated biological activities of propolis include antimicrobial, antioxidant, and antineoplastic effects [6,7,8,9,10,11].

Additionally, propolis exhibits a wide range of other bioactivities, including antiviral effects against emerging viruses such as SARS-CoV-2, HSV-1 and Chikungunya [12,13,14,15], as well as immunomodulatory, anti-inflammatory, antihypertensive, hypocholesterolemic, neuroprotective, and neurobehavioral effects [16,17,18,19,20,21,22,23,24,25,26]. Its composition is highly dependent on the botanical sources available to bees, resulting in significant chemical variability across geographic regions [27,28,29].

The chemical diversity of Brazilian propolis remains incompletely explored, particularly in underrepresented biomes such as the Pampa region in southern Brazil. Previous studies have described hundreds of compounds in propolis, including phenolic acids, flavonoids, terpenoids, and fatty acids [27,28,29], highlighting its chemical complexity. Despite this, most studies have focused on phenolic-rich propolis types, while samples with alternative chemical profiles remain poorly characterized.

Polyphenols are frequently suggested to be the potentially active compounds in antioxidant and antineoplastic activity [30,31]. However, the chemical composition of propolis varies both geographically and seasonally; thus, each propolis has particular nutraceutic and biological potentials. Therefore, it would not be correct to attribute its potential to a single cluster of compounds, nor would it be correct to attribute identical properties for distinct propolis [27]. For example, regarding the effect on the immune system, some propolis can develop pro-inflammatory effects and other anti-inflammatory effects [16]. In Brazil, this chemical diversity has been extensively documented, particularly for green and red propolis, which are the most studied and commercially explored types [32].

Green propolis, predominantly derived from *Baccharis dracunculifolia*, is characterized by a high content of phenolic compounds, especially Artepillin-C, and has been associated with strong antioxidant and antimicrobial properties [33,34,35]. Red propolis, originating mainly from *Dalbergia ecastophyllum*, exhibits a distinct chemical profile rich in isoflavonoids, which contributes to its potent biological activities, including anticancer effects [10,11,36]. These two types of propolis have shaped the current understanding that phenolic compounds are the primary drivers of propolis bioactivity [30,31].

This knowledge context raises important questions regarding the relationship between chemical composition and biological activity in propolis.

Among these biological activities, propolis has potential efficacy against hematological malignancies, such as leukemia, which has emerged as a highly significant field of study [37,38]. Leukemia remains a major global health challenge due to drug resistance, disease relapse, and the severe side effects associated with conventional chemotherapy [39].

Consequently, there is an urgent need to identify novel, selective natural agents capable of inducing apoptosis in leukemic cells while sparing healthy tissue. Although polyphenols and volatile components from green and red propolis have shown promising cytotoxic and pro-apoptotic effects in leukemia models [40,41], the antileukemic potential of alternative propolis profiles from unexplored ecosystems remains unknown, which raises crucial questions regarding how distinct chemical profiles influence specific biological mechanisms.

In this context, the southern region of Brazil, particularly the Pampa biome, represents an underexplored source of propolis diversity. Despite being one of the largest honey-producing regions in the country, this area faces economic and environmental challenges that highlight the need for diversification and value addition in apiculture [42,43,44,45,46].

Therefore, the present study aims to characterize a novel type of Brazilian propolis, herein referred to as “amber propolis”, originating from the Pampa biome. Using an integrated analytical approach combining GC-MS and LC-MS/MS, we investigated its chemical composition and evaluated its antimicrobial, antioxidant and antileukemic activities. Furthermore, these biological profiles were compared with those of the well-established Brazilian green and red propolis, providing new insights into how distinct chemical compositions dictate specific propolis bioactivity and offering a new high-value natural resource for nutraceutical evaluation.

## 2. Results

### 2.1. Chemical Characterization of Amber Propolis by Gas Chromatography with Mass Spectrometric Detection (GC-MS)

The volatile chemical composition of the amber propolis extracts (Samples A and B) was comprehensively characterized by GC-MS analysis. By applying a strict library search quality threshold (Similarity Index, SI > 80), high-confidence volatile constituents were successfully annotated and distributed across distinct chemical classes, as summarized in Table 1. The full chromatographic and mass spectral metadata for all detected peaks are detailed in Tables S2 and S3 (Supplementary Material).

The volatile profile of both samples (A and B) was characterized by a strong predominance of terpenoid compounds, although qualitative and quantitative discrepancies in the individual distribution of chemical families were observed between the two samples.

GC-MS analysis identified 69 compounds in Sample A and 62 compounds in Sample B of amber propolis, with terpenoids representing the predominant class (72% and 77%, respectively). Approximately 50% of the compounds were shared between both samples, resulting in a total of 99 distinct compounds identified across the two years and 42% of the compounds annotated between samples A and B are shared with Red and Green propolis. The chromatographic profiles of both samples exhibited a consistent chemical pattern, with similar predominant peaks (Figure S1). This shared volatile core was composed almost entirely of sesquiterpenoids, including highly stable markers such as trans-nerolidol (5.77% in A; 5.12% in B), globulol (5.03% in A; 5.88% in B) and spathulenol (0.48/2.62% in A; 5.32% in B). A major unresolved peak observed at approximately 42 min likely corresponds to co-eluting compounds. Another prominent peak at approximately 50 min was tentatively assigned to a tetracyclic structure; however, due to low spectral similarity (75%), it was excluded to ensure annotation reliability.

Comparative analysis revealed limited overlap with reference propolis types. Sample A shared 11 compounds with red propolis and 6 with green propolis, while Sample B shared 6 and 5 compounds with red and green propolis, respectively. Representative shared compounds included 6-camphenol, germacrene D, and oleic acid. The complete list of molecules detected by GC-MS, alongside a detailed comparison with literature data for green and red propolis and their respective citations, is presented in Table S1.

### 2.2. Analysis of the In Vitro Antioxidant Properties

**Figure 1:**
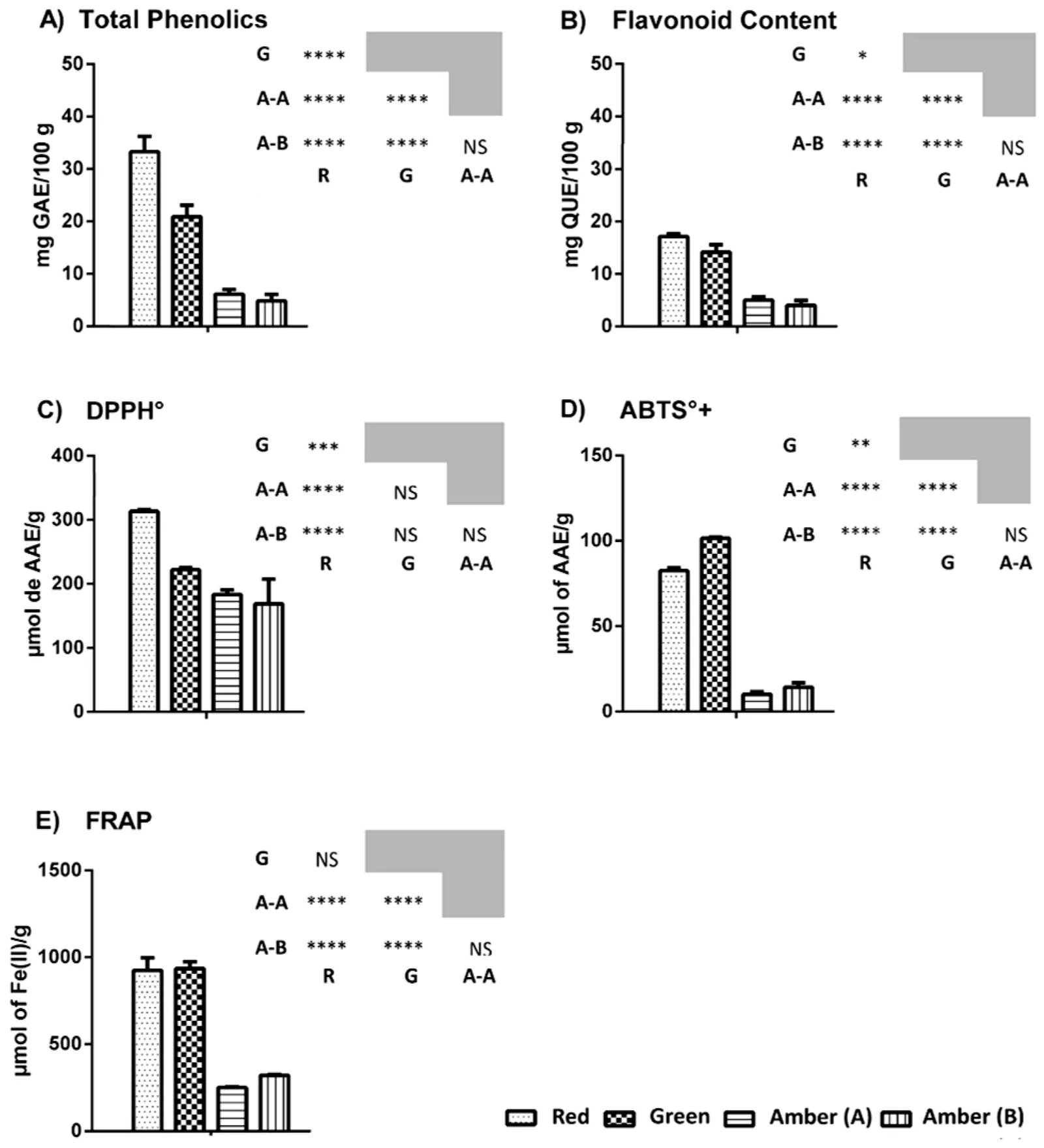
Total phenolic and flavonoid contents and in vitro antioxidant capacity of propolis extracts. (**A**) Total phenolic content; (**B**) total flavonoid content; (**C**) DPPH° radical scavenging activity; (**D**) ABTS°+ radical scavenging activity; and (**E**) Ferric Reducing Antioxidant Power (FRAP). (R) red propolis; (G) green propolis; (A-A) amber propolis sample A; (A-B) amber propolis sample B. Statistical significance is indicated by asterisks: * *p* ≤ 0.05, ** *p* ≤ 0.01, *** *p* ≤ 0.001, **** *p* ≤ 0.0001; NS: non-significant difference.

The evaluation of antioxidant properties revealed that amber propolis contains significantly lower concentrations of total phenolics and flavonoids than red and green propolis (approximately four-fold and two-fold lower, respectively) (Figure 1A,B). Regarding DPPH radical scavenging activity, no significant statistical difference was observed between the red propolis, green and the B sample of amber propolis; these three extracts exhibited higher activity than the sample A (Figure 1C). Furthermore, analysis of ABTS°+ radical scavenging and Ferric Reducing Antioxidant Power (FRAP) showed comparable effects between the green and red propolis, both of which presented antioxidant capacities approximately two-fold higher than those of amber propolis (Figure 1D,E).

### 2.3. Analysis of Cell Viability

**Figure 2:**
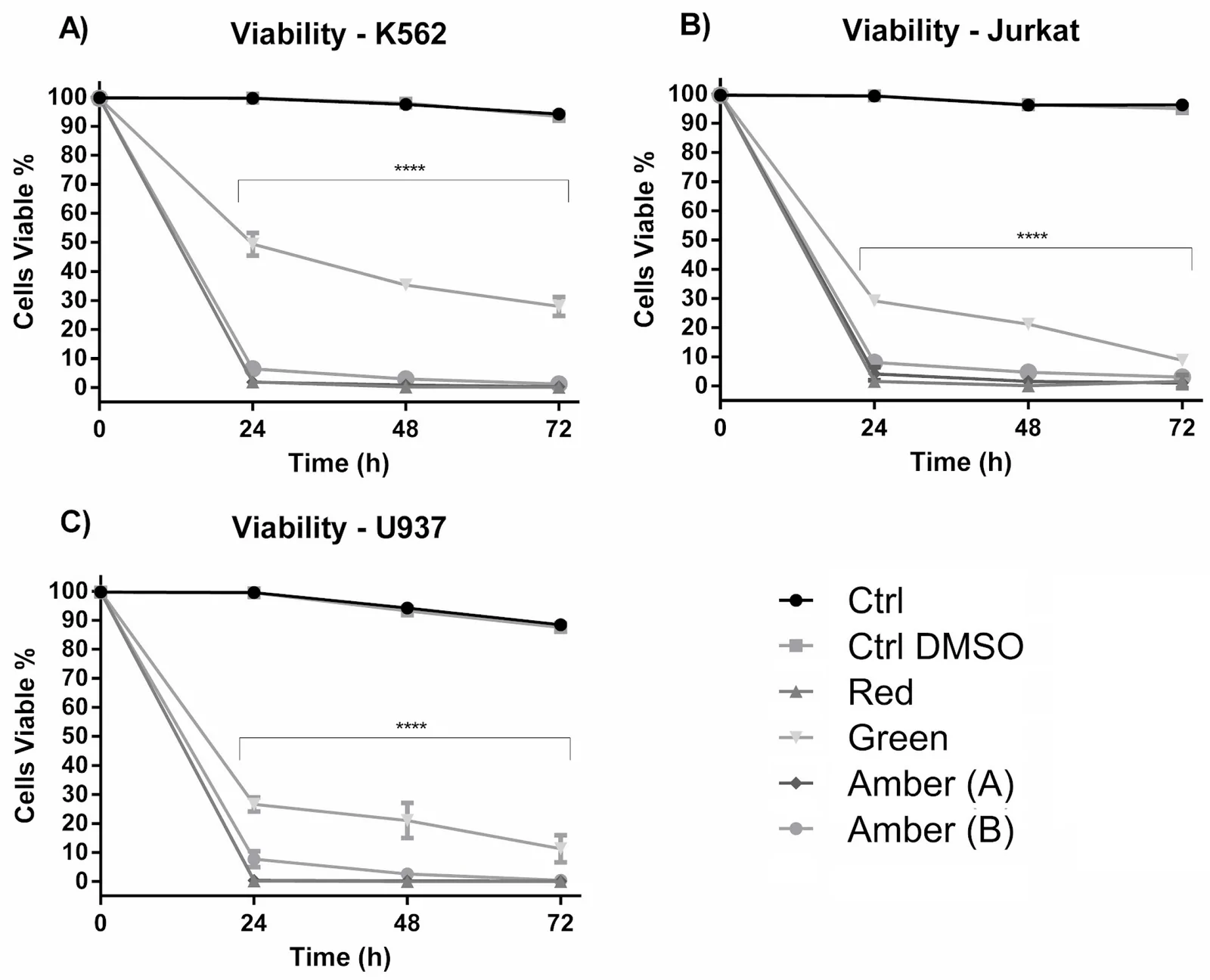
Cytotoxic effects of amber, green and red propolis extract on K562 (**A**), Jurkat (**B**) and U937 (**C**) cell lines. Cells were treated with the different propolis at 100 μg/mL concentration for 24, 48 and 72 h. Data are expressed as Mean ± SEM. The asterisks represent statistical significance: **** *p* ≤ 0.0001.

Treatment of K562, Jurkat and U937 cells with different types of propolis (100 µg/mL) significantly reduced cell viability after 24 h. Both amber propolis (samples A and B) and red propolis had similar cytotoxic effects, with no significant statistical differences observed between them (*p* > 0.05), inducing over 90% cell death across all three cell lines 24 h. In contrast, green propolis showed a lower citotoxic effect (*p* ≤ 0.0001 compared to both amber and red propolis), though its activity remained significant relative to the negative control (*p* ≤ 0.0001) (Figure 2A–C).

**Figure 3:**
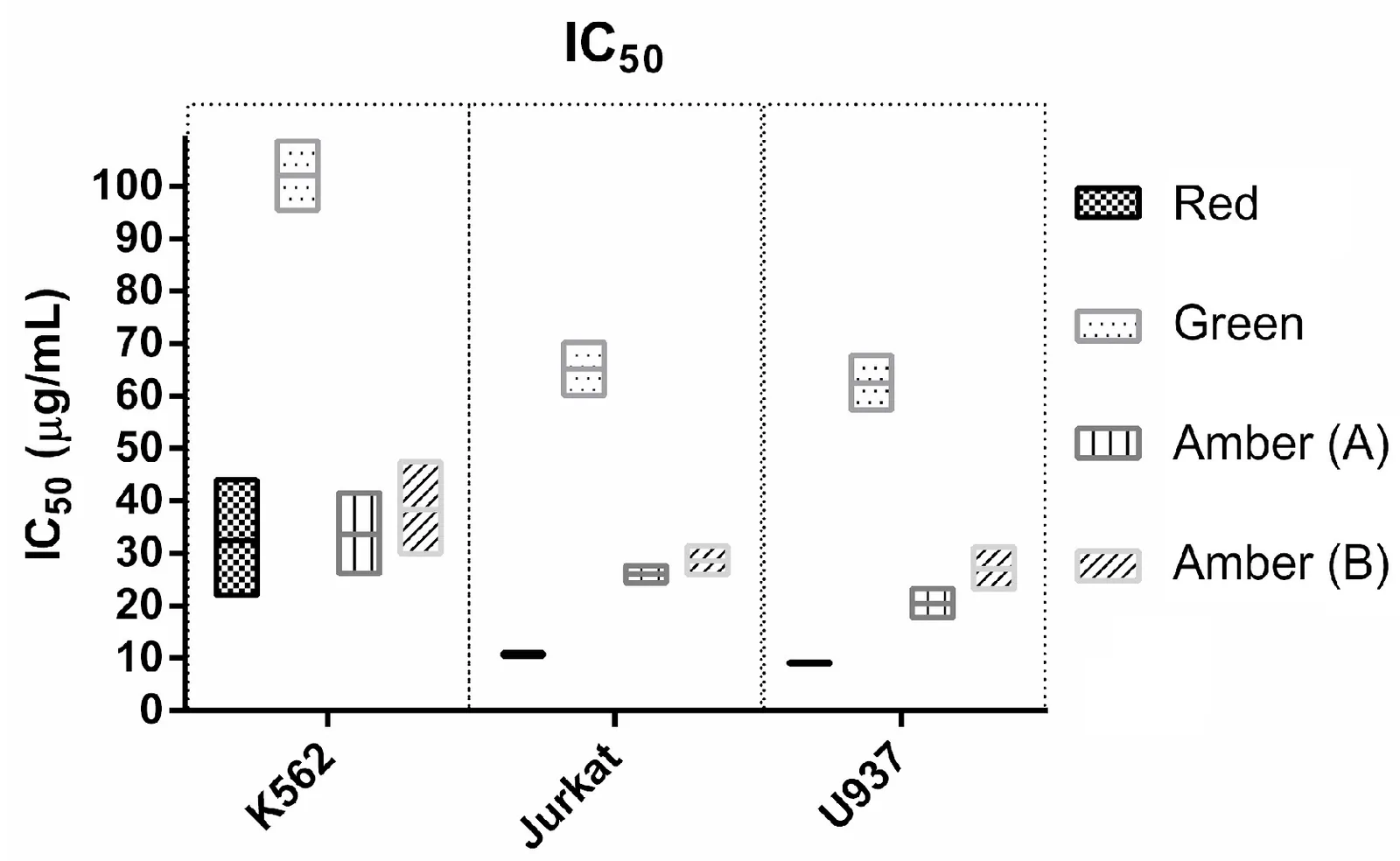
Mean of inhibitory concentration (IC_50_) values of amber, green and red propolis extract were determined by treating the K562, Jurkat and U937 cell lines with different extract concentrations (10–100 μg/mL) for 24 h. Red propolis: 31.12 μg/mL (K562), 10.68 μg/mL (Jurkat), 9.08 μg/mL (U937). Green propolis: 101.90 μg/mL (K562), 64.94 μg/mL (Jurkat), 62.29 μg/mL (U937). Amber propolis (Sample A): 32.97 μg/mL (K562), 26.01 μg/mL (Jurkat), 20.28 μg/mL (U937). Amber propolis (Sample B): 37.73 μg/mL (K562), 28.52 μg/mL (Jurkat), 26.85 μg/mL (U937).

Furthermore, we determined the half-maximal inhibitory concentration (IC_50_) of the different propolis extracts across the three cell lines. For K562 cells, no significant statistical differences were observed when comparing red propolis with amber propolis, with both presenting IC_50_ values of about 30 µg/mL, green propolis presenting IC_50_ values approximately three-fold higher (*p* ≤ 0.0001). Jurkat and U937 cell lines did not show statistical differences regarding the IC_50_ dose within each treatment; however, there were significant differences between the propolis treatments. Red propolis exhibited the lowest IC_50_ dose (approximately 10 µg/mL), while amber propolis showed a dose of approximately 25 µg/mL; notably, only the sample B of amber propolis showed statistical difference when compared to red propolis (*p* ≤ 0.05 in both cell lines). The green propolis dose was approximately 65 µg/mL, showing statistically significant differences when compared to red and amber propolis (*p* ≤ 0.0001 in both of the cell lines). Regarding the effect across cell lines, both red and green propolis had significantly lower IC_50_ values for U937 and Jurkat cells than for K562 cells (*p* ≤ 0.01 and *p* ≤ 0.0001, respectively); nevertheless, amber propolis showed no statistical difference in its effect on the different strains suggesting a more uniform cytotoxic potential. The IC_50_ values of each propolis in the different cell lines are shown in Figure 3.

### 2.4. Induction of Apoptosis by Propolis

**Figure 4:**
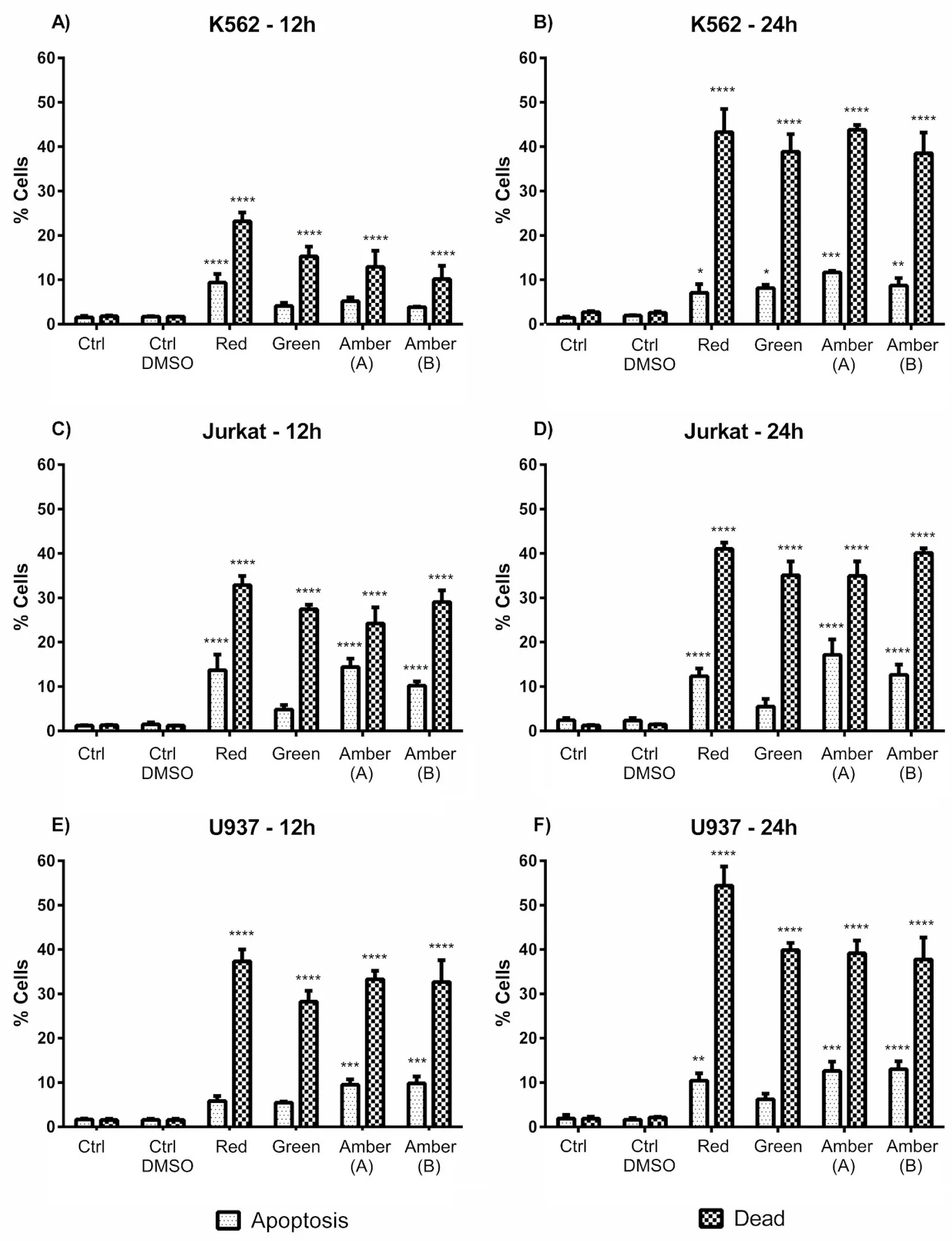
Effect of propolis on apoptotic cell death in K562 (**A**,**B**) Jurkat (**C**,**D**) and U937 (**E**,**F**) cell lines. The cells were treated for 24 h at the concentration values defined by IC_50_. Data are expressed as Mean ± SEM. The asterisks represent statistical significance: * *p* ≤ 0.05, ** *p* ≤ 0.01, *** *p* ≤ 0.001, **** *p* ≤ 0.0001.

The treatment of leukemic cell lines with different types of propolis at their half-maximal concentration (IC_50_) showed significant cell death compared to the control, beginning at 12 h of treatment in all tested cell lines (Figure 4A,C,E). In K562 cells, significant apoptosis was only observed after 12 h of treatment with red propolis, whereas green and amber propolis induced apoptotic events after 24 h (Figure 4A,B). Treatment with green propolis did not induce statistically significant apoptosis in the Jurkat or U937 cell lines at either the 12 or 24 h time points (Figure 4C–F). In contrast, treatment of Jurkat cells with red and amber propolis (samples A and B) induced significant apoptosis at both 12 and 24 h (Figure 4C,D). In U937 cells only amber propolis (samples A and B) induced significant apoptosis after 12 h of treatment (Figure 4E,F); after 24 h, red propolis also showed significant induction of apoptosis (Figure 4F). Supplementary Figure S1 shows representative flow cytometry plots, illustrating the gates used for the discrimination of the different cell groups.

### 2.5. Effect of Propolis Treatment on Cell Cycle

**Figure 5:**
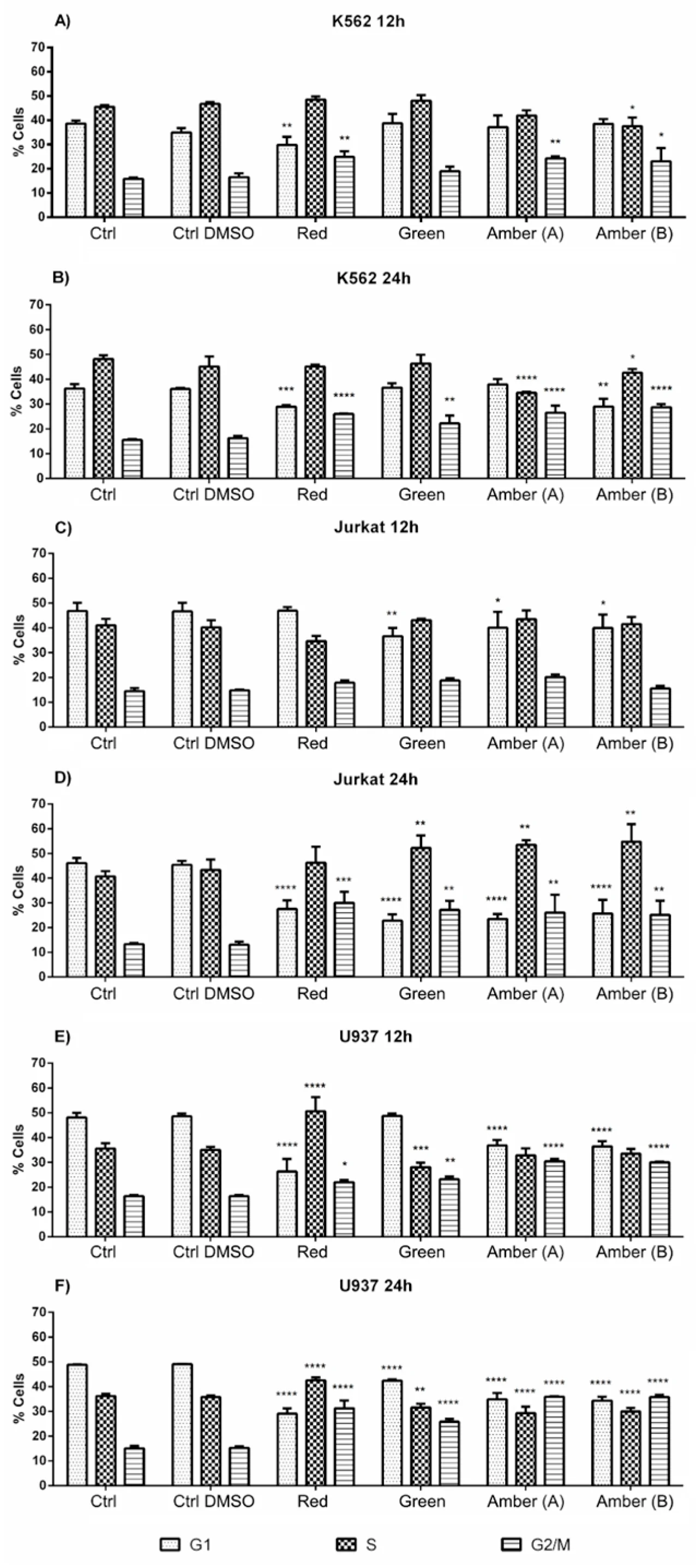
Effect of propolis on the cell cycle of cell lines K562 (**A**,**B**), Jurkat (**C**,**D**) and U937 (**E**,**F**). The cells were treated for 24 h at the concentration values defined by IC_50_ and analyzed at 12 and 24 h. Data are expressed as Mean ± SEM. The asterisks represent statistical significance: * *p* ≤ 0.05, ** *p* ≤ 0.01, *** *p* ≤ 0.001, **** *p* ≤ 0.0001.

The cell cycle analysis by flow cytometry revealed that all propolis types (used at IC_50_ dose) caused significant cell cycle arrest in the G2/M phase after 24 h of treatment in comparison with the controls (Figure 5B,D,F). G2/M arrest was observed in K562 cells after 12 h of treatment with red and amber propolis, but not with green propolis (Figure 5A). In Jurkat cells, no propolis extract caused G2/M arrest at the 12 h time point (Figure 5C). In contrast, in U937 cells, all tested propolis samples induced G2/M arrest beginning at 12 h of treatment (Figure 5E). Supplementary Figure S2 shows representative histograms of the cell cycle distribution for all propolis treatments.

### 2.6. Antibacterial Effect of Propolis

**Figure 6:**
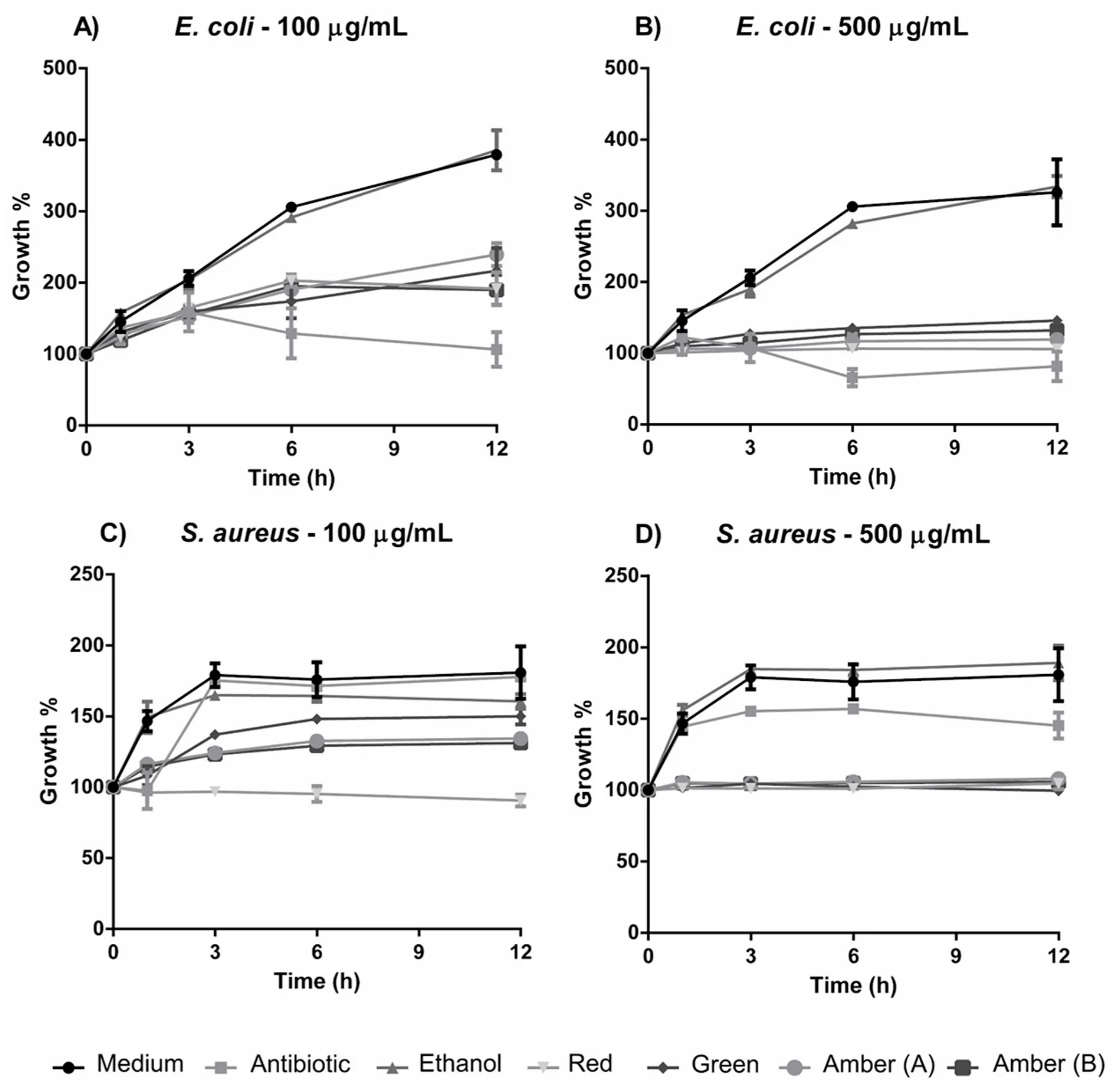
Propolis extracts effect in *Escherichia coli* growth at 100 µg/mL (**A**) and 500 µg/mL (**B**) concentrations and in *Staphylococcus aureus* growth at 100 µg/mL (**C**) and 500 µg/mL (**D**) concentrations. The absorbance measured at 600 nm during 12 h, at 0, 1, 3, 6, 12 h intervals. Data are expressed as Mean ± SEM.

The effect of propolis on *Escherichia coli* and *Staphylococcus aureus* growth was studied over 12 h of treatment at concentrations of 100 µg/mL and 500 µg/mL. In E. coli, a dose of 100 µg/mL resulted in a reduction in bacterial growth beginning at 3 h of treatment, with no significant statistical differences observed between the propolis types (Figure 6A). At this concentration, the positive control (penicillin/streptomycin solution) exhibited a significantly stronger inhibitory effect than the propolis extracts (*p* ≤ 0.01); however, at 500 µg/mL all propolis types effectively inhibited bacterial growth (Figure 6B).

In *S. aureus*, all three types of propolis showed superior antibacterial activity compared to the antibiotic solution, even at the lowest concentration tested, starting from 3 h of treatment (*p* ≤ 0.0001). Red propolis completely inhibited bacterial growth at 100 µg/mL. Both amber propolis samples did not show significant differences between them, but they exhibited a significantly higher inhibitory effect than green propolis (*p* ≤ 0.01). Nevertheless, neither of the amber samples achieved total growth inhibition at this concentration, unlike red propolis (*p* ≤ 0.0001) (Figure 6C). Treatment at 500 µg/mL with any of the propolis tested resulted in complete inhibition of bacterial growth (Figure 6D).

### 2.7. Chemical Profile of Amber Propolis Obtained from LC-MS/MS Analyses

**Figure 7:**
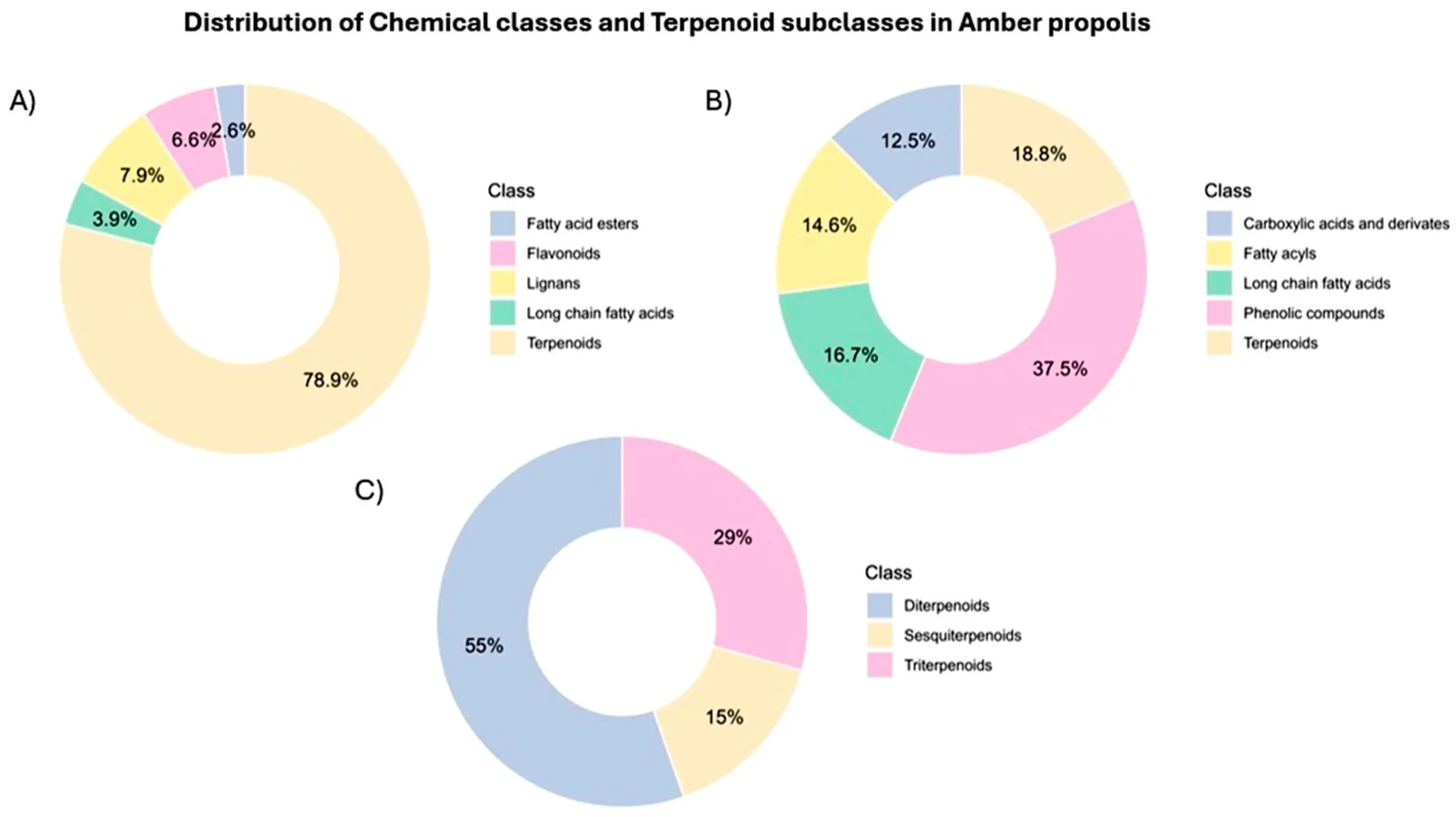
Distribution of chemical classes and terpenoid subclasses in amber propolis. (**A**) General chemical classes predicted in positive ionization mode; (**B**) General chemical classes predicted in negative ionization mode; (**C**) Detailed classification of the terpenoid subclass. Data were obtained by LC-MS/MS analysis via SIRIUS software (v. 5.8.6.0) using the CANOPUS tool (only annotations with confidence scores > 70% were included).

LC-MS/MS analysis of Sample C, performed in both positive and negative ionization modes and processed using SIRIUS (v. 5.8.6.0), resulted in the detection of 462 molecular features (250 in positive mode and 212 in negative mode). All detected features are reported in the Supplementary Material (Tables S1 and S2). Following a confidence-based curation workflow, 146 compounds were retained as high-confidence annotations, based on SIRIUS and CSI:FingerID (Tanimoto) scores ≥ 70%, combined with manual validation of ionization adducts through consistency between experimental *m*/*z* values and calculated exact masses. This curated dataset is presented in Supplementary Tables S3 and S4. In the raw datasets (Supplementary Tables S1 and S2), structural annotations (SMILES) were only reported for compounds with Tanimoto scores ≥ 70%, ensuring that structural representations were restricted to higher-confidence predictions. The distribution of chemical classes predicted by the CANOPUS tool demonstrated a predominance of terpenoid-related compounds (78.9%) in positive mode (Figure 7A), corroborating the chemical profile observed in GC-MS analysis and the non-phenolic constituents possessing the highest annotation identity are presented in Table 2. While phenolic compounds formed the majority class (37.5%), a substantial proportion of terpenoids (18.8%) was still observed (Figure 7B). The predominant classes in both ionization modes are represented by the 146 compounds classified as high-confidence (scores ≥ 70%), according to Supplementary Tables S4 and S5.

The phenolic profile obtained by LC–MS/MS revealed a set of predominant phenolic compounds in amber propolis, including flavonoids with chemical modifications and phenolic derivatives, encompassing glycosylated forms such as vicenin-1, vicenin-2, and isoviolanthin; these major phenolic compounds identified via LC–MS/MS are detailed in Table 3. Importantly, LC–MS/MS allowed the characterization of these diverse structural forms despite the relatively low total phenolic and flavonoid concentrations determined quantitatively in amber propolis when compared to reference green and red propolis.

## 3. Discussion

The present study describes a unique variety of propolis originating from the Pampa biome in Rio Grande do Sul, which our group has denominated as “amber propolis.” Historically, Brazilian propolis research has focused on green propolis (Cerrado biome, Minas Gerais) and red propolis (mangroves, Alagoas), while varieties from the southernmost state were often reported to have lower antimicrobial activity [42]. This research group proposes the term “amber propolis” to differentiate this highly active variety from the “yellow propolis” previously cataloged [42], which is frequently associated with low biological interest. By adopting this nomenclature, we highlight a distinct chemical fingerprint that challenges the secondary status of southern Brazilian propolis [43,47,48] and establishes its potential for therapeutic valuation.

The chemical profile of amber propolis, processed via SIRIUS and the CANOPUS tool, revealed a high diversity of mono-, di-, sesqui-, and triterpenoids. In the positive ionization mode, terpenoids represented the dominant chemical class, accounting for 78.9% of the annotated molecular features, whereas in the negative ionization mode they constituted 18.8% of the features. Conversely, phenolic compounds and flavonoids emerged as a major group in the negative ionization mode, representing 37.5% of the total annotations. It is crucial to clarify that these percentages refer to the proportion of the total number of identified chemical features within each separate ionization distribution rather than their absolute mass.

This strong terpenoid-centric profile was further substantiated and detailed through the GC-MS characterization of the volatile fractions (Table 1). The gas chromatography analysis aligned perfectly with the LC-MS/MS data, unveiling a complex matrix composed of 84 high-confidence volatile constituents (SI > 80) predominantly classified as monoterpenoids and sesquiterpenoids.

Regarding the biological assays, it is worth noting that all direct comparisons and IC_50_ evaluations were conducted using stock solutions prepared strictly from gravimetrically standardized dry matter weights, ensuring physical uniformity across samples. While amber propolis naturally exhibits a lower total phenolic yield compared with the green and red reference varieties, its substantial bioactivity demonstrates that normalizing biological results solely by phenolic content would artificially bias the interpretation. Propolis activity is driven by a complex matrix of nutraceutical compounds; in the case of amber propolis, the highly abundant terpenoids identified in our screenings may play a critical role in the overall biological response, compensating for the lower abundance of conventional polyphenols.

Given the geographical context, the proximity to Schinus species and Eucalyptus plantations suggests that these may be candidates for botanical sources. This hypothesis is supported by the identification of low level of eucalyptol in one amber propolis sample, a well-known marker for Eucalyptus [49], and triterpenoids such as lupenone and ursolic acid, which are characteristic constituents of the Schinus genus [50,51]; however, this hypothesis remains unconfirmed and warrants further direct investigation.

Despite its lower phenolic abundance compared to red and green varieties, amber propolis maintained high DPPH° scavenging activity. This suggests that the predominant terpenoids contribute significantly to the antioxidant effects. The mechanisms involved likely include membrane stabilization and the modulation of lipid peroxidation, as previously described for various terpene classes [52,53], as well as indirect redox regulation. Among the predominant flavonoids identified in amber propolis, the C-glycosylflavones vicenin-1, vicenin-2, and isoviolanthin stand out due to their distinct distribution patterns compared to other well-characterized Brazilian propolis types. Notably, none of these compounds have been reported in red propolis, suggesting a divergent metabolic origin. Vicenin-2 has been previously identified in green propolis samples from Lavras-MG, Cabo Verde-BA, and Mirabela-MG, as well as in a black propolis variety from Pirenópolis-GO [54]. In contrast, reports of vicenin-1 in propolis remain extremely scarce, with its occurrence documented primarily in propolis from stingless bees [55]. Strikingly, to the best of our knowledge, there are no prior reports detailing the presence of isoviolanthin in any propolis type. This unprecedented finding highlights isoviolanthin as a novel chemical entity within this matrix, positioning it as a potential specific biomarker for amber propolis that warrants targeted investigation in future studies.

The comprehensive profiling of the phenolic fraction via LC–MS/MS highlights the features that are present in relatively high abundance within the matrix, but its performance can be conditioned by fundamental factors inherent to the analytical approach. While LC–MS/MS provides broad structural screening, its detection capacity strictly depends on ionization efficiency, fragmentation behavior, and database annotation [56,57]. In particular, the ionization of phenolic compounds is strongly influenced by their chemical structure as well as the method used for elution of the sample in LC-MS/MS analyses, and certain flavonoid aglycones may exhibit low ionization efficiency or produce insufficient fragmentation patterns for confident annotation in untargeted workflows [58].

Moreover, flavonoids in natural products are frequently present as glycosylated or otherwise modified derivatives rather than free aglycones [59,60]. This is consistent with the LC–MS/MS results, which revealed the presence of glycosylated flavonoids such as vicenin-1/2 and isoviolanthin. Therefore, while LC–MS/MS is highly efficient for mapping major constituents and structural diversity, in untargeted workflows, long-term targeted approaches, such as validated HPLC-DAD methods, represent a logical next step. Such methodologies will be instrumental in future studies to precisely track and quantify these phenotypic markers across different seasons and consecutive years, establishing the seasonal chemical stability required for geographical indication.

Regarding its antileukemic potential, amber propolis demonstrated remarkable potency with IC_50_ values comparable to red propolis in K562 cells (~30 µg/mL). A particularly compelling finding was the uniform cytotoxicity across K562, Jurkat, and U937 strains, suggesting the triggering of conserved signaling pathways. One such pathway involves the transcription factor NF-KB, an essential survival mediator in K562 cells. We have previously demonstrated that pharmacological inhibition of NF-KB induces apoptosis in leukemia cells through the ROS-mitochondria pathway [61]. The presence of ursolic acid in amber propolis provides a molecular basis for this effect, as it is a documented inhibitor of IKBα kinase and p65 phosphorylation [62], correlating with the G2/M phase arrest and rapid induction of apoptosis (12 h in U937 cells) observed in our assays.

Finally, amber propolis exhibited a significant antibacterial effect against *S. aureus*, surpassing the green variety. We emphasize that the agar diffusion method may underestimate the potential of such terpenoid-rich varieties due to the poor diffusion of apolar molecules, such as mono- and triterpenes, through polar media, as recently reported by our group [63]. On the other hand, it is important to note that this specific antimicrobial assay was structured as an exploratory kinetic screening using two fixed concentrations, rather than a standardized multi-dose MIC determination as recommended by EUCAST/CLSI guidelines. Due to the inherent resinous nature of the crude extracts, which causes significant optical interference and light scattering, it was methodologically mandatory to include specific cell-free blank controls for each sample to ensure baseline accuracy.

While this targeted design represents a methodological limitation for direct clinical benchmarking, the positive results obtained successfully validate the dynamic bacteriostatic potential of amber propolis. Consequently, these findings establish a solid scientific baseline that opens the field for future bioprospecting studies dedicated exclusively to a comprehensive antimicrobial evaluation against an expanded panel of microorganisms. Ultimately, this study serves as a foundational step for the scientific validation and geographic recognition of amber propolis, aiming to increase its added value and support the economic and sustainable trajectory of this unique genetic resource from the Pampa biome.

## 4. Materials and Methods

### 4.1. Reagents

RPMI 1640 medium (Applichem, Darmstadt, Germany), Fetal Bovine Serum, penicillin and streptomycin (Gibco, São Paulo, SP, Brazil) were used for cell culture. The bacterial culture medium was composed of tryptone (Neogen, Lansing, MI, USA) and HiMedia yeast extract (Acumedia, Mumbai, India). YOPRO^®^-1 iodide (Invitrogen, Carlsbad, CA, USA), Propidium Iodide (≥94.0% purity), Folin–Ciocalteu radical 2,2-diphenyl-1-picrylhydrazyl (DPPH°), diammonium salt 2 2-azinobis-[3-ethyl-benzotiazolin-6-sulfonic acid] sodium acetate, 2,4,6-tris (2-pyridyl)-s-triazine (TPTZ), Aluminum chloride, potassium persulfate, ammonium sulfate, iron (II) hexahydrate and dimethyl sulfoxide (DMSO) were purchased from Vetec Fine Chemicals LTD (Rio de Janeiro, RJ, Brazil). All other chemicals used in this work have analytical grade.

### 4.2. Origin of Propolis

Brazilian propolis samples, herein referred to as “amber propolis”, were produced by *Apis mellifera* and collected over two consecutive years (sample A and B) for biological assays and chemical analyses (GC-MS). An additional sample (sample C) was used for LC-MS/MS analysis. All samples were obtained from the experimental apiary of the Universidade Federal do Pampa (UNIPAMPA), located in São Gabriel, Rio Grande do Sul, Brazil, during the spring seasons of 2022, 2023 and 2024, respectively. For comparison, commercial raw propolis samples from Alagoas (red propolis) and Minas Gerais (green propolis) were used. In all regions, bee populations are characterized as Africanized *Apis mellifera*.

### 4.3. Propolis Extract Production

Ethanolic extracts of propolis (EEP) were prepared by dissolving raw propolis in ethanol (30% *w*/*v*) under constant stirring for 7 days at room temperature. After extraction, samples were stored at −20 °C overnight to promote wax precipitation, followed by centrifugation at 1600× *g* for 10 min. The supernatant was collected, filtered, and ethanol was evaporated using a vacuum concentrator (Eppendorf Concentrator Plus, Hamburg, Germany) at 60 °C until complete solvent removal and the achievement of a constant dry weight. The yield of each completely dried crude extract was accurately determined gravimetrically using an analytical balance, resulting in extraction yields of 328.2 mg/g for Sample A, 407.4 mg/g for Sample B, and 384.0 mg/g for Sample C. The dried extracts were then resuspended either in dimethyl sulfoxide (DMSO) for cell-based assays (EEP/DMSO) or in absolute ethanol for antimicrobial assays (EEP/EtOH), both at a final concentration of 10% (*w*/*v*). For chemical analyses (GC-MS and LC-MS/MS), extracts were solubilized in methanol (EEP/MeOH, 10% *w*/*v*). All samples were filtered through 0.45 µm membranes prior to analysis.

### 4.4. Analyses of Propolis Through GC-MS

GC–MS analysis was performed using a Shimadzu GCMS-QP2010 Plus system (Shimadzu Corporation, Kyoto, Japan) equipped with an RTX-5MS capillary column (30 m × 0.25 mm i.d., 0.25 µm film thickness), consisting of 5% diphenyl and 95% dimethylpolysiloxane (Restek Corporation, Bellefonte, PA, USA). Samples were injected in splitless mode at an injector temperature of 250 °C. Helium was used as carrier gas at a constant flow rate of 0.95 mL/min. The oven temperature program was set as follows: initial temperature of 50 °C (5 min), increased to 300 °C at 10 °C/min, and held for 30 min. The ion source and transfer line temperatures were maintained at 280 °C. Retention index (RI) values reported by the NIST08 library were utilized as complementary filters for compound annotation, ensuring that the theoretical elution order matched the experimental chromatographic behavior and aiding in the exclusion of false-positive library matches. Additionally, the obtained data were compared with literature data published for green and red propolis [64,65].

### 4.5. Analysis of the In Vitro Antioxidant Properties

In vitro antioxidant properties were analyzed spectrophotometrically in 96-well plates using an EnSpire multimode plate reader (PerkinElmer, Waltham, MA, USA), following the methodology previously described by our group for the study of eucalyptus honey [66].

#### 4.5.1. DPPH Radical Scavenging Assay

DPPH radical scavenging activity was determined according to [66], with minor modifications. Briefly, 100 µL of DPPH solution (300 µM in ethanol) were mixed with 50 µL of propolis extract (0.1 g/mL), and the final volume was adjusted to 300 µL with ethanol. After incubation for 45 min at room temperature in the dark, absorbance was measured at 517 nm. Ascorbic acid was used as a positive control. Results were expressed as µmol of ascorbic acid equivalents (AAE) per 100 g of propolis.

#### 4.5.2. ABTS°+ Radical Scavenging Assay

ABTS radical cation scavenging activity was determined as described by [67], with modifications. The ABTS•+ solution was generated by reacting 7 mM ABTS with 2.5 mM potassium persulfate and incubating the mixture in the dark. A total of 200 µL of ABTS•+ solution were mixed with 10 µL of propolis extract (0.1 g/mL), and absorbance was measured at 734 nm after 10 min. Ascorbic acid (1 mM) was used as a positive control. Results were expressed as µmol AAE per g of propolis.

#### 4.5.3. Ferric Reducing Antioxidant Power (FRAP) Assay

Ferric reducing antioxidant power (FRAP) was evaluated according to [68], with adaptations for propolis samples. Propolis extracts (0.1 g/mL) were mixed with 270 µL of FRAP reagent composed of 0.3 M acetate buffer (pH 3.6), 10 mM TPTZ solution, and FeCl_3_·6H_2_O. Samples were incubated at 37 °C for 30 min, and absorbance was measured at 595 nm. A standard curve was prepared using ammonium iron(II) sulfate (100–2000 µM). Results were expressed as µmol Fe(II) equivalents per g of propolis.

### 4.6. Determination of the Total Phenolics Compounds

Total phenolic content was determined using the Folin–Ciocalteu method [69], with minor modifications. Propolis extracts (0.5 mg/mL) were mixed with Folin–Ciocalteu reagent and 15% sodium carbonate solution. After incubation in the dark for 2 h, absorbance was measured at 760 nm. Gallic acid was used as a standard (10–300 µg/mL), and results were expressed as mg of gallic acid equivalents (GAE) per 100 g of propolis.

### 4.7. LC-MS/MS Analysis of Amber Propolis Ethanolic Extract

LC–MS/MS analyses of amber propolis extract were performed at the Rio de Janeiro Center for Biomolecule Mass Spectrometry (CEMBIO-RJ). Samples were solubilized in methanol and diluted to 1 mg/mL in MeOH:H_2_O (1:1, *v*/*v*). Analyses were conducted using a Shimadzu Nexera X2 UHPLC system coupled to a Maxis Impact ESI-Q-TOF mass spectrometer (Bruker, Bremen, Germany). Aliquots of 10 µL (negative mode) and 5 µL (positive mode) were injected onto a Hypersil C18 column (150 mm × 2.1 mm, 3 µm; Thermo Fisher Scientific, Waltham, MA, USA). The mobile phase consisted of solvent A (water) and solvent B (methanol), with a flow rate of 0.2 mL/min and the following gradient: 0 min (10% B), 9 min (70% B), 9.5 min (100% B), 13 min (100% B), 14 min (10% B), and 19 min (stop). Mass spectrometry was performed using electrospray ionization (ESI) in both positive and negative modes. Data acquisition was carried out in data-dependent acquisition mode (DDA/AutoMS), with fragmentation of up to five precursors per cycle, within an *m*/*z* range of 50–1500, and an acquisition rate of 1.5 Hz. Internal calibration was performed using 100 µM sodium formate in water/acetonitrile (1:1, *v*/*v*). Raw data were exported in .mzXML format for further analysis.

### 4.8. Chemical Profile Analysis of LC-MS/MS Data from Amber Propolis

LC–MS/MS data were analyzed using SIRIUS software (version 5.8.6.0), widely used in metabolomics and natural product research [70]. The .mzXML files from positive and negative ionization modes were processed using the integrated tools: SIRIUS (molecular formula prediction based on fragmentation patterns), ZODIAC (Bayesian refinement of molecular formulas), CSI:FingerID (structure prediction and database matching using PubChem, GNPS, and COCONUT), and CANOPUS (compound class prediction) [70,71,72,73,74]. Compounds were annotated based on SIRIUS scores (formula confidence) and TANIMOTO scores (structure similarity). All detected features were reported individually, including structural isomers and oxidized derivatives, as they represent distinct MS/MS signals. Chemical classification followed CANOPUS predictions, which may assign structurally related metabolites to different subclasses. Therefore, no deduplication based on biosynthetic similarity was performed.

### 4.9. Cell Culture and Treatments

K562(BCRJ Code: 0126), Jurkat (BCRJ Code: 0125), and U937(BCRJ Code: 0242) cell lines were obtained from the The cell lines were purchased from the Rio de Janeiro Cell Bank (BCRJ) and cultured in RPMI 1640 medium supplemented with 10% fetal bovine serum, 100 U/mL penicillin, and 100 µg/mL streptomycin. Cells were maintained at 37 °C in a humidified atmosphere with 5% CO2. Cells were seeded at an initial density of 0.5 × 105 cells/mL and allowed to stabilize for 24 h prior to treatment. Propolis extracts (red, green, amber A, and amber B) were diluted in DMSO (10% *w*/*v*) and applied at different concentrations depending on the assay. Cells treated with DMSO alone were used as negative controls. All experiments were performed in triplicate.

### 4.10. Analysis of Cell Viability and Determination of IC_50_ Dose

Cell viability was assessed using propidium iodide (PI) exclusion assay. Cells were seeded in 24-well plates and treated with propolis extracts (100 µg/mL) for 72 h. For IC_50_ determination, cells were treated with increasing concentrations (10–100 µg/mL) for 24 h. After treatment, cells were incubated with PI (1.25 µg/mL) and analyzed by flow cytometry, acquiring 10,000 events per sample. Cell viability was determined based on PI exclusion, and IC_50_ values were calculated using nonlinear regression.

### 4.11. Apoptosis Assay

Apoptosis was evaluated using the YO-PRO-1/propidium iodide staining method. Cells were treated with propolis extracts at IC_50_ concentrations for 24 h. After treatment, cells were incubated with 100 nM YO-PRO-1 and 150 nM PI for 5 min and analyzed by flow cytometry. A total of 30,000 events were acquired and analyzed to distinguish viable, apoptotic, and necrotic cells. The assay was performed without the washing procedure with PBS. After incubation, 30.000 gated events were analyzed by flow cytometry in FL1-H (YOPRO) vs. FL3-H (PI) density plot.

### 4.12. Cell Cycle Analysis

Cell cycle distribution was analyzed using PI staining as described by [75], with modifications. Cells were treated with propolis extracts at IC_50_ concentrations, lysed, and stained with PI-containing buffer. After 5 min, cells were analyzed by flow cytometry by collecting 5.000 gated events, in slow mode, FL2-H vs. FL2-A density plot. Later, the cell cycle was analyzed in FL2-A histograms by FlowJo X v.0.7 software.

### 4.13. Antimicrobial Activity

*Escherichia coli* and *Staphylococcus aureus* strains were grown in Luria–Bertani medium containing 1% Tryptone, 0.5% NaCl and 0.5% yeast extract to examine the effect of propolis extracts in their growth. Initially, bacteria cultures were incubated at 37 °C under constant agitation (180 RPM) until the optical density of approximately 0.4 was reached for *E. coli* and 0.2 for *S. aureus* (600 nm in a SP-22 spectrophotometer Biospectro, Curitiba, PR, Brazil). A total of 100 µL of bacterial culture were transferred to 96-well plates containing 100 µL of LB medium. The plates were incubated at 37 °C with agitation (50 RPM) and the absorbance measured at 600 nm during 12 h, at 0, 1, 3, 6, 12 h intervals, in a spectrophotometer EnSpire^®^ multimode (PerkinElmer, Waltham, MA, USA). For negative controls and blanks we replicated the treatment conditions, but without bacteria. As positive controls we used ampicillin and streptomycin.

### 4.14. Statistical Analysis

Results are expressed as mean ± standard deviation (SD) of at least three independent experiments. Statistical analyses were performed using two-way ANOVA followed by Tukey’s or Dunnett’s post hoc tests, as appropriate. Differences were considered statistically significant at *p* ≤ 0.05. IC_50_ values were calculated using nonlinear regression with a sigmoidal dose–response model.

## 5. Conclusions

This study provides the first comprehensive characterization of amber propolis from the Brazilian Pampa, revealing a terpenoid-dominant chemical fingerprint that sets it apart from traditional green and red varieties. Our findings contribute to the understanding that the biological activities of propolis extend beyond its phenolic content, highlighting the functional importance of the terpenoid fraction. Regarding its functional properties, the investigated matrix demonstrated the following.

Antioxidant activity: The amber propolis exhibits radical scavenging activity, which is mediated by its specific phenolic and flavonoid fractions despite their lower overall concentrations compared to reference green and red propolis.

Antileukemic effects: The matrix induces apoptosis within 12 h in Jurkat and U937 cell lines and 24 h in K562 cell line, alongside G_2_/M cell cycle arrest across all three evaluated lineages. This uniform cytotoxic profile indicates a distinct pattern of activity, suggesting the potential involvement of different biochemical pathways than those typically modulated by green and red propolis.

Antimicrobial activity: The samples display inhibitory activity against *S. aureus*; notably, at the specific concentrations evaluated (100 and 500 μL/mL), the amber propolis demonstrated a greater inhibitory effect than both green propolis.

In conclusion, amber propolis emerges as a valuable natural resource with biotechnological and nutraceutical potential. Beyond its pharmaceutical interest, its multi-target biological activities position it as a promising functional food ingredient from the Pampa biome. These results establish a scientific baseline for the geographical indication and economic valorization of propolis from Rio Grande do Sul, providing a new alternative for the pharmaceutical and food industries.

## 6. Patents

Specific data regarding the biological activities and molecular mechanisms of the amber propolis extracts reported in this manuscript are part of a patent application filed with the Brazilian National Institute of Industrial Property (INPI) under registration number BR1020250035200.

## Figures and Tables

**Table 1:** Volatile chemical composition and relative abundance (%) of amber propolis Samples A and B determined by GC-MS analysis.

Compound Name	Theoretical RI (NIST) *	Sample A Area (%)	Sample A SI	Sample B Area (%)	Sample B SI	Chemical Class
α-Phellandrene	969	—	—	0.38	94	Monoterpenoid
α-Pinene	948	—	—	0.81	96	Monoterpenoid
β-Myrcene	958	—	—	0.24	91	Monoterpenoid
Decane	1015	—	—	0.21	96	Aliphatic hydrocarbon
o-Cymene	1042	—	—	0.15	93	Monoterpenoid (Aromatic)
Limonene	1018	—	—	0.48	92	Monoterpenoid
Eucalyptol	1059	—	—	0.08	81	Monoterpenoid
γ-Terpinene	998/897	—	—	0.13	92/93	Monoterpenoid
trans-Sabinene hydrate	1041	—	—	0.20	92	Monoterpenoid
Sabinol derivative	1085/943	—	—	01.01	82/97	Monoterpenoid
Pinocarveol	1131	—	—	2.40	94	Monoterpenoid
Verbenol	1136/1191	2.50	84	4.43	87/84	Monoterpenoid
Phellandral	1175	—	—	0.18	85	Monoterpenoid
Camphenilone derivative	1114	—	—	0.67	89	Monoterpenoid
Verbenyl ethyl ether	1184	—	—	0.53	81	Monoterpenoid derivative
Perillaldehyde isomer	1175	—	—	0.25	83	Monoterpenoid
Lavandulal derivative	1074	—	—	0.38	80	Monoterpenoid
Myrtenol	1191	—	—	3.22	94	Monoterpenoid
Verbenone	1119	—	—	1.51	96	Monoterpenoid
cis-Carveol	1206	—	—	0.61	91	Monoterpenoid
Methoxy-methylbutane	565	—	—	0.47	81	Aliphatic ether
Carvone	1190	—	—	0.12	91	Monoterpenoid
Pinanediol	1276	—	—	0.33	80	Monoterpenoid
Bornyl acetate	1277	—	—	0.49	94	Monoterpenoid ester
Perillyl alcohol	1261	—	—	0.20	90	Monoterpenoid
Pinene oxide derivative	1020	—	—	0.20	86	Monoterpenoid
Phthalic acid	1620	—	—	0.57	93	Aromatic acid/Plasticizer
p-Menth-8-ene-1,2-diol	1346	2.32	91	—	—	Monoterpenoid
α-Cubebene	1344	0.75	96	0.73/0.12	93/84	Sesquiterpenoid
α-Copaene	1221	0.72	94	0.43	93	Sesquiterpenoid
trans-3(10)-Caren-2-ol	1346	28.14	82	—	—	Monoterpenoid
Epoxy-terpenyl acetate	1131	1.40	80	—	—	Monoterpenoid ester
β-Elemene isomer	1398	1.18	91	—	—	Sesquiterpenoid
1-Butylpyrrole	975	—	—	0.56	83	Alkaloid/Nitrogen compound
Ledene	1386	—	—	0.98	90	Sesquiterpenoid
α-Gurjunene	1419	0.33	91	0.21	91	Sesquiterpenoid
Campholenic aldehyde	1155	4.20/0.70	90/88	0.11	85	Monoterpenoid
α-Cedrene	1393	05.06	82	—	—	Sesquiterpenoid
Dimethylnonadienol	1247	01.05	84	0.57	85	Aliphatic alcohol
Methylhexadecenal isomer	1843	—	—	0.11	81	Fatty aldehyde
Myrtenol derivative	1191	10.43	81	—	—	Monoterpenoid
Guaiene isomer	1461	1.22	89	—	—	Sesquiterpenoid
Cyclopropanemethanol derivative	1299	13.17	85	—	—	Terpenoid derivative
Lavandulal isomer	1092	01.06	86	—	—	Monoterpenoid
β-Selinen-derivative	1469	1.83	94	—	—	Sesquiterpenoid
γ-Gurjunene	1490	2.13	91	2.16	91	Sesquiterpenoid
Aromadendrene	1386	—	—	0.45	87	Sesquiterpenoid
Biphenylene derivative	1443	—	—	0.34	83	Polycyclic hydrocarbon
Neral	1174	—	—	0.41	87	Monoterpenoid
β-Himachalene derivative	1456	—	—	1.97	94	Sesquiterpenoid
α-Bulnesene	1490	—	—	0.15	81	Sesquiterpenoid
α-Selinene isomer	1435	—	—	4.30	84	Sesquiterpenoid
Cadinene isomer	1469	—	—	1.88	88	Sesquiterpenoid
Selina-6-en-4-ol	1593	5.99	84	—	—	Sesquiterpenoid
α-Calacorene	1547	1.16	89	1.39	89	Sesquiterpenoid
trans-Nerolidol	1564	5.77	96	5.12	93	Sesquiterpenoid
Ledene oxide	1293	01.07	85	—	—	Sesquiterpenoid
Spathulenol	1536	0.48/2.62	94/82	5.32	94	Sesquiterpenoid
Globulol	1530	5.03/1.37	94/90	5.88/1.31	91/92	Sesquiterpenoid
Viridiflorol isomer	1530	1.37	90	1.31	92	Sesquiterpenoid
Cadinol isomer	1580	2.11	84	1.50	83	Sesquiterpenoid
α-Cadinol	1580	1.70	90	1.26	92	Sesquiterpenoid
9-Methoxycalamenene	1686	1.35	84	—	—	Sesquiterpenoid derivative
p-Menth-3-ene-1,2,8-triol	1574	2.43	91	—	—	Monoterpenoid
α-Amorphenol	1647	4.21	83	—	—	Sesquiterpenoid
Cadala-1(10),3,8-triene	1423	1.38	86	2.86	88	Sesquiterpenoid
Eudesmol derivative	1690	0.98/1.94	83/82	—	—	Sesquiterpenoid
Phytol isomer	2192	3.23	80	—	—	Diterpenoid
Aromadendrene oxide	1462	3.80	82	—	—	Sesquiterpenoid
Geranylgeraniol	2192	8.42	91	—	—	Diterpenoid
Dihydroxyfarnesyl acetate	2103	07.06	87	—	—	Sesquiterpenoid ester
Cembradiol isomer	2400	1.24	82	—	—	Diterpenoid
Methyl hexadecanoate	1878	4.11	93	—	—	Fatty acid ester
n-Hexadecanoic acid	1968	2.25	92	—	—	Fatty acid
Megastigmatrienone	1454	0.94	81	—	—	Norisoprenoid
Methyl octadecanoate	2077	2.14	92	—	—	Fatty acid ester
Oleic acid	2175	1.96	92	—	—	Fatty acid
Ethyl oleate	2185	0.96	93	—	—	Fatty acid ester
Lanosterol	2882	8.47	82	28.48	84	Triterpenoid
Urs-12-en-28-al	2873	2.78	83	—	—	Triterpenoid
24-Methylenecycloartanol	2834	2.33	81	—	—	Triterpenoid
Humulane-1,6-dien-3-ol	1757	6.71	81	—	—	Sesquiterpenoid
Isoaromadendrene epoxide	1281	—	—	0.59	84	Sesquiterpenoid
Heneicosane	2109	—	—	0.69	94	Aliphatic hydrocarbon

Note: Compounds are listed in chronological order of elution based on their chromatographic behavior. Data include only high-confidence annotations with a library search similarity index (SI > 80). Common names are used to enhance readability; full raw datasets, structural details, and original library metadata are provided in Tables S5 and S6 (Supplementary Material). “—” indicates that the compound was not detected or was below the screening quality threshold. * Theoretical RI (NIST) values are listed for literature comparison and informational purposes only, and were not utilized as an active compound identification or filtering criterion.

**Table 2:** Chemical constituents of South Brazilian amber propolis (Sample C) identified by LC-MS/MS analysis.

Compound	Molecular Formula	SIRIUS Score	*m*/*z*	Adduct	Chemical Class
Ferruginol	C_20_H_28_O	100%	285.22	[M+H]+	Diterpenoids
Communic acid	C_20_H_30_O_2_	100%	303.23	[M+H]+	Diterpenoids
Isocupressic acid	C_20_H_32_O_3_	100%	321.24	[M+H]+	Diterpenoids
Pinifolic acid	C_20_H_32_O_4_	100%	337.24	[M+H]+	Diterpenoids
Grandiflorenic acid	C_20_H_28_O_2_	100%	301.22	[M+H]+	Diterpenoids
Sclareol	C_20_H_36_O_2_	100%	309.28	[M+H]+	Diterpenoids
Hydroxylated abietic acid	C_20_H_36_O_4_	100%	321.24	[M−H]−	Diterpenoids
Cedrol derivative	C_20_H_28_O	99.97%	283.21	[M−H]−	Diterpenoids
Ursolic acid	C_30_H_46_O_3_	100%	455.35	[M+H]+	Triterpenoids
Uvaol	C_30_H_50_O_2_	99.99%	443.39	[M+H]+	Triterpenoids
Moronic acid	C_30_H_46_O_3_	94.98%	453.34	[M−H]−	Triterpenoids
Rotundic acid	C_30_H_48_O_5_	99.85%	487.34	[M−H]−	Triterpenoids
β-amyrin (β-amirona)	C_30_H_48_O	100%	425.38	[M+H]+	Triterpenoids
Matairesinol	C_20_H_22_O_6_	99.91%	376.18	[M+H]+	Lignans
Arctigenin-like lignan	C_21_H_24_O_6_	97.52%	390.19	[M+H]+	Lignans
Bursehernin	C_21_H_22_O_6_	99.98%	388.18	[M+H]+	Lignans
Isopicropodophyllin	C_23_H_24_O_8_	95.56%	429.15	[M+H]+	Lignans
Caffeic acid phenethyl ester (CAPE)	C_17_H_16_O_4_	98.74%	283.10	[M−H]−	Phenolic acids
Ferulic acid derivative	C_18_H_26_O_3_	100%	289.18	[M−H]−	Phenolic compounds
Biphenyltricarboxylic acid	C_16_H_10_O_8_	100%	329.03	[M−H]−	Phenolic compounds
Oleic acid	C_18_H_34_O_2_	100%	281.25	[M−H]−	Fatty acids
Hydroxy-eicosanoic acid	C_20_H_40_O_3_	100%	327.29	[M−H]−	Fatty acids
Gondoic acid	C_20_H_38_O_2_	99.98%	309.28	[M−H]−	Fatty acids
Arachidic acid	C_20_H_40_O_2_	99.91%	311.29	[M−H]−	Fatty acids
Urushiol II	C_21_H_34_O_2_	99.53%	317.25	[M−H]−	Phenolic lipids
Oleamide	C_18_H_35_NO	100%	282.28	[M+H]+	Lipids
Batilol	C_21_H_44_O_3_	100%	345.34	[M+Na]+	Monoacylglycerols

Note: Data represent selected compounds obtained by LC-MS/MS in both ionization modes (negative and positive) using ESI-Q-TOF. Compounds were selected based on chemical relevance, annotation confidence, and their overall contribution to the chemical profile. For the complete compound annotations, see Tables S6 and S7 in the Supplementary Material.

**Table 3:** Phenolic and flavonoid profile of amber propolis identified by LC–MS/MS analysis.

Phenolic Profile of Amber Propolis					
Compound Annotation	Molecular Formula	SIRIUS Score	*m*/*z*	Adduct	Chemical Class
Caffeic acid phenethyl ester (CAPE)	C_17_H_16_O_4_	98.74%	283.10	[M−H]−	Phenolic acid derivative
Ferulic acid derivative	C_18_H_26_O_3_	100%	289.18	[M−H]−	Phenolic acid derivative
Dimethoxyphenyl derivative	C_21_H_28_O_4_	99.00%	343.19	[M−H]−	Phenolic derivative
Urushiol II	C_21_H_34_O_2_	99.53%	317.25	[M−H]−	Phenolic lipid
Phenylpropanoid derivative	C_15_H_24_O_3_	99.88%	251.17	[M−H]−	Phenylpropanes
Apigenin	C_15_H_10_O_5_	100%	269.05	[M−H]−	Flavonoid
Isokaempferide	C_16_H_12_O_6_	88.43%	299.00	[M−H]−	Flavonoid
Prunetin	C_16_H_12_O_5_	99.87%	283.06	[M−H]−	Flavonoid
Lethedocin derivative	C_18_H_16_O_7_	99.17%	343.08	[M−H]−	Flavonoid
Vicenin-2	C_27_H_30_O_15_	94.00%/98.00%	593.15	[M−H]−/[M+H]+	Flavonoid C-glycoside
Vicenin-1	C_26_H_28_O_14_	97.80%	565.16	[M+H]+	Flavonoid C-glycosides
Isoviolanthin	C_27_H_30_O_14_	99.53%	579.17	[M+H]+	Flavonoid C-glycosides
Isoschaftoside 2″-ferulate	C_36_H_36_O_17_	97.62%	741.20	[M+H]+	Flavonoid C-glycosides
Alpinone	C_16_H_14_O_5_	99.97%	287.09	[M+H]+	Flavonoid

Note: Data include only compounds with SIRIUS and CSI:FingerID (Tanimoto) scores ≥ 70% and consistent adduct assignment. Compound identifications are putative, based on MS/MS fragmentation patterns and database matching. For the complete annotation dataset, see Tables S6 and S7 in the Supplementary Material.

## Data Availability

The data supporting the chemical characterization, mass spectrometry features, and biological activities evaluated in this study are fully available within the article and its accompanying Supplementary Material files (Tables S1–S5).

## Supplementary Materials

The following supporting information can be downloaded at: https://www.mdpi.com/article/10.3390/molecules31183143/s1, Figure S1. Representative gas chromatography-mass spectrometry (GC-MS) total ion chromatograms (TIC) of amber propolis volatile profiles for Sample A (top) and Sample B (bottom); Figure S2: Representative flow cytometry histograms of cell cycle distribution in K562, Jurkat, and U937 leukemia cell lines treated for 12 and 24 h with different propolis types analyzed by propidium iodide (PI) fluorescence intensity; Figure S3. Representative flow cytometry gating strategy for cell viability and apoptosis analysis in K562 cell line after 24 h of treatment, utilizing YO-PRO-1 (FL1-H) and propidium iodide (PI, FL3-H) staining to define live, apoptotic, and dead cell populations in control (Ctrl) and amber propolis (Amber A and Amber B) samples; Table S1: Qualitative comparative profile of chemical constituents identified by GC-MS in South Brazilian amber propolis (Samples A and B) and their correlation with reference data for Brazilian red and green propolis; Table S2. Detailed peak integration report, retention times (RT), area percentages, and NIST library identification parameters obtained by GC-MS analysis for amber propolis Sample A; Table S3. Detailed peak integration report, retention times (RT), area percentages, and NIST library identification parameters obtained by GC-MS analysis for amber propolis Sample B; Table S4: Putative annotation of chemical constituents in South Brazilian amber propolis by LC-MS/MS (positive ionization mode), including chemical formulas, adducts, SMILES notations, SIRIUS/Tanimoto similarity scores, and compound classifications; Table S5: Putative annotation of chemical constituents in South Brazilian amber propolis by LC-MS/MS (negative ionization mode), including chemical formulas, adducts, SMILES notations, SIRIUS/Tanimoto similarity scores, and compound classifications; Table S6: LC-MS/MS chemical profiling and putative annotation of amber propolis constituents (positive ionization mode) featuring retention times, Tanimoto/SIRIUS scoring, and chemical ontology classification; Table S7: LC-MS/MS chemical profiling and putative annotation of amber propolis constituents (negative ionization mode) featuring retention times, Tanimoto/SIRIUS scoring, and chemical ontology classification.

## References

[1] Burdock G.A. (1998). Review of the biological properties and toxicity of bee propolis. Food Chem. Toxicol..

[2] Castaldo S., Capasso F. (2002). Propolis, an old remedy used in modern medicine. Fitoterapia.

[3] Kuropatnicki A.K., Szliszka E., Krol W. (2013). Historical aspects of propolis research in modern times. Evid. Based Complement. Altern. Med..

[4] Zabaiou N., Fouache A., Trousson A., Baron S., Zellagui A., Lahouel M., Lobaccaro J.-M.A. (2017). Biological properties of propolis extracts: Something new from an old product. Chem. Phys. Lipids.

[5] Zullkiflee N., Taha H., Usman A. (2022). Therapeutic benefits of propolis in modern medicine. Molecules.

[6] Machado B.A., Silva R.P.D., de Abreu Barreto G., Costa S.S., da Silva D.F., Brandão H.N., da Rocha J.L.C., Dellagostin O.A., Henriques J.A.P., Umsza-Guez M.A. (2016). Chemical composition and biological activity of extracts obtained by supercritical extraction. PLoS ONE.

[7] Lazo G.L., Lemes D.C., Pauletti P.M., Bastos J.K., Ambrósio S.R., Veneziani R.C.S., Pires R.H. (2025). Analysis of the antimicrobial activity of propolis: A narrative review of in-vitro studies. An. Acad. Bras. Cienc..

[8] Zhao L., Pu L., Wei J., Li J., Wu J., Xin Z., Gao W., Guo C. (2016). Antioxidant activity of propolis. Int. J. Environ. Res. Public Heal..

[9] Zhong-Yong J., ZHi-qing D., Li-qiong X., Poorasadollah E., Shirvani S. (2024). Impact of propolis supplementation on inflammatory biomarkers: A meta-analysis and systematic review of randomized controlled clinical trials. Prostaglandins Other Lipid Mediat..

[10] da Silva Frozza C.O., Santos D.A., Rufatto L.C., Minetto L., Scariot F.J., Echeverrigaray S., Pich C.T., Moura S., Padilha F.F., Borsuk S. (2017). Antitumor activity of Brazilian red propolis fractions against Hep-2 cancer cell line. Biomed. Pharmacother..

[11] Altabbal S., Athamnah K., Rahma A., Wali A.F., Eid A.H., Iratni R., Al Dhaheri Y. (2023). Propolis: A detailed insight of its anticancer molecular mechanisms. Pharmaceuticals.

[12] Vynograd N., Vynograd I., Sosnowski Z. (2000). Comparative multi-centre study of the efficacy of propolis. Phytomedicine.

[13] Negri G., Duarte-Almeida J.M., Figueiredo C.A., de Toledo-Piza A.R., Tonelli F.C.P., Barbosa T.F., Mendonça R.Z. (2024). Antiviral Activity of Red Propolis Against Herpes Simplex Virus-1. Braz. J. Pharm. Sci..

[14] Berretta A.A., Silveira M.A.D., Cóndor Capcha J.M., De Jong D. (2020). Propolis and Its Potential against SARS-CoV-2 Infection Mechanisms and COVID-19 Disease. Biomed. Pharmacother..

[15] Kontogiannis T., Dimitriou T.G., Didaras N.A., Mossialos D. (2022). Antiviral Activity of Bee Products. Curr. Pharm. Des..

[16] Conti B.J., Santiago K.B., Búfalo M.C., Herrera Y.F., Alday E., Velazquez C., Hernandez J., Sforcin J.M. (2015). Modulatory effects of propolis samples from Latin America on cytokine production. J. Pharm. Pharmacol..

[17] Bueno-Silva B., Kawamoto D., Ando-Suguimoto E.S., Casarin R.C.V., Alencar S.M., Rosalen P.L., Mayer M.P.A. (2017). Brazilian red propolis effects on peritoneal macrophage activity. J. Ethnopharmacol..

[18] Bhatti N., Hajam Y.A., Mushtaq S., Kaur L., Kumar R., Rai S. (2024). A review on dynamic pharmacological potency and multifaceted biological activities of propolis. Discov. Sustain..

[19] Mishima S., Inoh Y., Narita Y., Ohta S., Sakamoto T., Araki Y., Suzuki K.-M., Akao Y., Nozawa Y. (2005). Identification of caffeoylquinic acid derivatives from Brazilian propolis. Bioorg. Med. Chem..

[20] Qu H., Cao L., Wen Z., Li C., Xiao M. (2025). The effect of propolis supplementation on blood pressure: A systematic review and meta-analysis of controlled trials. Minerva Cardiol. Angiol..

[21] Yu Y., Si Y., Song G., Luo T., Wang J., Qin S. (2011). Ethanolic Extract of Propolis Promotes Reverse Cholesterol Transport and the Expression of ATP-Binding Cassette Transporter A1 and G1 in Mice. Lipids.

[22] Bahari H., Shahraki Jazinaki M., Goudarzi K., Namkhah Z., Taheri S., Golafrouz H., Pahlavani N. (2025). Effects of propolis consumption on blood pressure, lipid profile and glycemic parameters in adults. Br. J. Nutr..

[23] Reis J.S.S., Oliveira G.B., Monteiro M.C., Machado C.S., Torres Y.R., Prediger R.D., Maia C.S.F. (2014). Anxiolytic-like effect of a Brazilian propolis. Phytomedicine.

[24] Nanaware S., Shelar M., Sinnathambi A., Mahadik K.R., Lohidasan S. (2017). Neuroprotective effect of Indian propolis in β-amyloid induced memory deficit. Biomed. Pharmacother..

[25] Sajjadi S.S., Bagherniya M., Soleimani D., Siavash M., Askari G. (2023). Effect of propolis on mood, quality of life, and metabolic profiles in subjects with metabolic syndrome. Sci. Rep..

[26] Zhu A., Wu Z., Zhong X., Ni J., Li Y., Meng J., Du C., Zhao X., Nakanishi H., Wu S. (2018). Brazilian Green Propolis Prevents Cognitive Decline into Mild Cognitive Impairment in Elderly People Living at High Altitude. J. Alzheimer’s Dis..

[27] Kasote D., Bankova V., Viljoen A.M. (2022). Propolis: Chemical diversity and challenges in quality control. Phytochem. Rev..

[28] Bankova V., Bertelli D., Borba R., Conti B.J., da Silva Cunha I.B., Danert C., Eberlin M.N., I Falcão S., Isla M.I., Moreno M.I.N. (2019). Standard methods for *Apis mellifera* propolis research. J. Apic. Res..

[29] Toreti V.C., Sato H.H., Pastore G.M., Park Y.K. (2013). Recent Progress of Propolis for Its Biological and Chemical Compositions and Its Botanical Origin. Evid. Based Complement. Altern. Med..

[30] Kurek-Górecka A., Kurek-Górecka A., Kłósek M., Pietsz G., Czuba Z.P., Kolayli S., Can Z., Balwierz R., Olczyk P. (2014). Structure and antioxidant activity of polyphenols derived from propolis. Molecules.

[31] Jain P.K., Parashar A.K., Shrivastava V. (2025). Exploring the health benefits and antioxidant properties of bioactive polyphenols. Discov. Food.

[32] Silva B.B., Rosalen P.L., Cury J.A., Ikegaki M., Souza V.C., Esteves A., Alencar S.M. (2007). Chemical composition of Brazilian red propolis. Evid. Based Complement. Altern. Med..

[33] Park Y.K., Park Y.K., Alencar S.M., Aguiar C.L. (2002). Botanical origin of Brazilian green propolis. Evid. Based Complement. Altern. Med..

[34] Park Y.K., Alencar S.M., Aguiar C.L. (2004). *Baccharis dracunculifolia*, the main botanical source of Brazilian green propolis. J. Sci. Food Agric..

[35] Shahinozzaman M., Basak B., Emran R., Rozario P., Obanda D.N. (2020). Artepillin C: A comprehensive review of its chemistry, bioavailability, and pharmacological properties. Fitoterapia.

[36] Daugsch A., Moraes C.S., Fort P., Park Y.K. (2008). Brazilian red propolis—Chemical composition and botanical origin. Evid. Based Complement. Altern. Med..

[37] Eom H.S., Lee E.J., Yoon B.S., Yoo B.S. (2010). Propolis inhibits the proliferation of human leukaemia HL-60 cells by inducing apoptosis through the mitochondrial pathway. Nat. Prod. Res..

[38] Campoccia D., Ravaioli S., Santi S., Mariani V., Santarcangelo C., De Filippis A., Montanaro L., Arciola C.R., Daglia M. (2021). Exploring the anticancer effects of standardized extracts of poplar-type propolis: In vitro cytotoxicity toward cancer and normal cell lines. Biomed. Pharmacother..

[39] Whiteley A.E., Price T.T., Cantelli G., Sipkins D.A. (2021). Leukaemia: A model metastatic disease. Nat. Rev. Cancer.

[40] Franchi G.C., Moraes C.S., Toreti V.C., Daugsch A., Nowill A.E., Park Y.K. (2012). Comparison of effects of the ethanolic extracts of brazilian propolis on human leukemic cells as assessed with the MTT assay. Evid. Based Complement. Altern. Med..

[41] Abubakar M.B., Abdullah W.Z., Sulaiman S.A., Ang B.S. (2014). Polyphenols as key players for the antileukaemic effects of propolis. Evid. Based Complement. Altern. Med..

[42] Park Y.K., Ikegaki M., Alencar S.M. (2000). Classificação das própolis brasileiras a partir de suas características fisico-químicas e propriedades biológicas. Mensagem Doce.

[43] Sawaya A.C., Cunha I.B., Marcucci M.C., de Oliveira Rodrigues R.F., Eberlin M.N. (2006). Brazilian propolis of Tetragonisca angustula and Apis mellifera. Apidologie.

[44] Wolff L.F., Gomes J.C.C. (2015). Beekeeping and agroecological systems for endogenous sustainable development. Agroecol. Sustain. Food Syst..

[45] Marcolin L.C., Lima L.R., De Oliveira Arias J.L., Berrio A.C.B., Kupski L., Barbosa S.C., Primel E.G. (2021). *Meliponinae* and *Apis mellifera* honey in southern Brazil. Food Chem..

[46] IBGE (2024). Instituto Brasileiro de Geografia e Estatística. https://sidra.ibge.gov.br.

[47] Park Y.K., Alencar S.M., Aguiar C.L. (2002). Própolis produzida no sul do Brasil, Argentina e Uruguai: Evidências fitoquímicas de sua origem vegetal. Ciênc. Rural.

[48] Ribeiro V.P., Mejia J.A.A., Rodrigues D.M., Alves G.R., De Freitas Pinheiro A.M., Tanimoto M.H., Bastos J.K., Ambrósio S.R. (2023). Brazilian Brown Propolis: An Overview About Its Chemical Composition, Botanical Sources, Quality Control, and Pharmacological Properties. Rev. Bras. Farmacogn..

[49] De Vincenzi M., Silano M., De Vincenzi A., Maialetti F., Scazzocchio B. (2002). Constituents of aromatic plants: Eucalyptol. Fitoterapia.

[50] Mügge F.L.B., Morlock G.E. (2023). Chemical and cytotoxicity profiles of 11 pink pepper (*Schinus* spp.) samples via non-targeted hyphenated high-performance thin-layer chromatography. Metabolomics.

[51] Perigo F., Haber L.L., Facanali R., Vieira M.A.R., Torres R.B., Bernacci L.C., Guimarães E.F., Baitello J.B., Sobral M.E.G., Quecini V. (2022). Essential Oils of Aromatic Plant Species from the Atlantic Rainforest Exhibit Extensive Chemical Diversity and Antimicrobial Activity. Antibiotics.

[52] Sikkema J., de Bont J.A., Poolman B. (1995). Mechanisms of toxicity of cyclic hydrocarbons to microorganisms. Microbiol. Rev..

[53] Grassmann J. (2005). Terpenoids as plant antioxidants. Vitam. Horm..

[54] Righi A.A., Negri G., Salatino A. (2013). Comparative chemistry of propolis from eight brazilian localities. Evid. Based Complement. Altern. Med..

[55] Cisilotto J., Sandjo L.P., Faqueti L.G., Fernandes H., Joppi D., Biavatti M.W., Creczynski-Pasa T.B. (2018). Cytotoxicity mechanisms in melanoma cells and UPLC-QTOF/MS_2_ chemical characterization of two Brazilian stingless bee propolis: Uncommon presence of piperidinic alkaloids. J. Pharm. Biomed. Anal..

[56] Stalikas C.D. (2007). Extraction, separation, and detection methods for phenolic acids and flavonoids. J. Sep. Sci..

[57] Wolfender J.-L., Marti G., Thomas A., Bertrand S. (2015). Current approaches and challenges for the metabolite profiling of complex natural extracts. J. Chromatogr. A.

[58] Cuyckens F., Claeys M. (2004). Mass spectrometry in the structural analysis of flavonoids. J. Mass Spectrom..

[59] Tsimogiannis D., Samiotaki M., Panayotou G., Oreopoulou V. (2007). Characterization of Flavonoid Subgroups and Hydroxy Substitution by HPLC-MS/MS. Molecules.

[60] Kachlicki P., Einhorn J., Muth D., Kerhoas L., Stobiecki M. (2008). Evaluation of glycosylation and malonylation patterns in flavonoid glycosides during LC/MS/MS metabolite profiling. J. Mass Spectrom..

[61] Zanotto-Filho A., Delgado-Cañedo A., Delgado-Cañedo A., Schröder R., Becker M., Klamt F., Moreira J.C.F. (2010). NF-κB inhibitors induce cell arrest and apoptosis. Cancer Lett..

[62] Shanmugam M.K., Dai X., Kumar A.P., Tan B.K.H., Sethi G., Bishayee A. (2013). Ursolic acid in cancer prevention and treatment: Molecular targets, pharmacokinetics and clinical studies. Biochem. Pharmacol..

[63] Roll R.J., Delgado-Cañedo A. (2025). Antimicrobial properties of propolis extracts: A closer look at glycolic solvent effects. Rev. FT.

[64] Alencar S.M., Oldoni T.L.C., Castro M.L., Cabral I.S.R., Costa-Neto C.M., Cury J.A., Rosalen P.L., Ikegaki M. (2007). Chemical composition and biological activity of a new type of Brazilian propolis: Red propolis. J. Ethnopharmacol..

[65] Nunes C.A., Guerreiro M.C. (2012). Characterization of Brazilian green propolis throughout the seasons by headspace GC/MS and ESI-MS. J. Sci. Food Agric..

[66] Cruz L.C., Ecker A., Dias R.S., Seeger R.L., Braga M.M., Boligon A.A., Martins I.K., Costa-Silva D.G., Barbosa N.V., Delgado-Cañedo A. (2016). Brazilian Pampa Bioma honey protects against mortality, locomotor deficits and oxidative stress. Neurochem. Res..

[67] Baltrušaitytė V., Venskutonis P.R., Čeksterytė V. (2007). Radical scavenging activity of different floral origin honey and beebread phenolic extracts. Food Chem..

[68] Benzie I.F.F., Strain J.J. (1996). The ferric reducing ability of plasma (FRAP). Anal. Biochem..

[69] Singleton V.L., Orthofer R., Lamuela-Raventós R.M. (1998). Analysis of total phenols and other oxidation substrates and antioxidants. Methods Enzymol..

[70] Dührkop K., Fleischauer M., Ludwig M., Aksenov A.A., Melnik A.V., Meusel M., Dorrestein P.C., Rousu J., Böcker S. (2019). SIRIUS 4: A rapid tool for turning tandem mass spectra into metabolite structure information. Nat. Methods.

[71] Dührkop K., Shen H., Meusel M., Rousu J., Böcker S. (2015). Searching molecular structure databases with tandem mass spectra using CSI:FingerID. Proc. Natl. Acad. Sci. USA.

[72] Ludwig M., Nothias L.-F., Dührkop K., Koester I., Fleischauer M., Hoffmann M.A., Petras D., Vargas F., Morsy M., Aluwihare L. (2020). ZODIAC: Database-independent molecular formula annotation. Anal. Chem..

[73] Hoffmann M.A., Nothias L.-F., Ludwig M., Fleischauer M., Gentry E.C., Witting M., Dorrestein P.C., Dührkop K., Böcker S. (2021). Assigning confidence to structural annotations from mass spectra with COSMIC. bioRxiv.

[74] Dührkop K., Nothias L.-F., Fleischauer M., Reher R., Ludwig M., Hoffmann M.A., Petras D., Gerwick W.H., Rousu J., Dorrestein P.C. (2021). Systematic classification of unknown metabolites using high-resolution fragmentation mass spectra. Nat. Biotechnol..

[75] Overton W.R., McCoy J.P. (1994). Reversing the Effect of Formalin on the Binding of Propidium Iodide to DNA. Cytometry.

