# Supplementary material for: South Brazilian Amber Propolis: Chemical Profile, and Antioxidant, Antimicrobial and Antileukemic Activities
Source: Molecules. 2026 Sep 8;31(18):3143. doi: 10.3390/molecules31183143 (PMC13609837; doi:10.3390/molecules31183143)
Supplement: Supplementary file 1 [file molecules-31-03143-s001.zip › Table S1.pdf]

Table S1. Qualitative comparative profile of chemical constituents identified by GC-MS in South Brazilian amber propolis (Samples A and B) and their correlation with reference data for Brazilian red and green propolis.

Compounds	Sample A	Sample B
**Terpenes**		
(E,E,E)-3,7,11,15-Tetramethylhexadeca-1,3,6,10,14-pentaene	✓	
(S)-cis-Verbenol	✓	✓
(z,e)-farnesol	✓	
1,6,10,14,18,22-tetracosahexaen-3-ol, 2,6,10,15,19,23-hexamethyl-, (all-e)-	✓	
4,8,13-Duvatriene-1,3-diol	✓	
4-Thujanol		✓
6-isopropenyl-4,8a-dimethyl-1,2,3,5,6,7,8,8a-octahydro-naphthalen-2-ol	✓	
7-tetracyclo[6.2.1.0(3.8)0(3.9)]undecanol, 4,4,11,11-tetramethyl	✓	
9-methoxycalamenene	✓	
Alloaromadendrene		✓
Alpha-Cadinol	✓R	✓R
Alpha-Calacorene	✓	✓
Alpha-Cubebene	✓R, G	✓G
Alpha-Guaiene	✓	✓
Alpha-Gurjunene	✓	✓

Alpha-Phellandrene		✓
Alpha-Pinene	✓R,G	✓R,G
Alpha-Thujene (3-Thujene)	✓	
Aromadendrene		✓G
Aromadendrene oxide	✓	
Beta-elemene	✓	
Beta-pinene	✓R,G	✓R,G
Beta-Pinene epoxide (2,10-Epoxypinane)		✓
Beta-Selinene	✓	
Beta-Thujene (2-thujene)	✓	
Betulin	✓	
Bicyclo[3.2.0]heptan-3-ol, 2-methylene-6,6-dimethyl-	✓	
Bicyclo[5.3.0]decane, 2-methylene-5-(1-methylvinyl)-8-methyl-		✓
Bornyl acetate	✓	✓
Cadala-1(10),3,8-triene	✓	✓
Cadinol	✓R	✓R
Camphenol, 6-		✓
Carvone		✓

Cis-Beta-Guainene	✓	
Cis-Carveol	✓	✓
Cis-Sabinol		✓
Copaene (Alpha-Copaene)	✓$_{R,G}$	✓$_{R,G}$
Cosmene		✓
Cubenol (10.beta.H-Cadin-4-en-1-ol)	✓	
Cyclo Sativene	✓	
D-carvone		✓
Delta-cadiene (Cadina-1(10),4-diene)	✓$_{R,G}$	✓$_{R,G}$
Delta-Guaiene		✓
Eucalyptol (Cineole)		✓$_{G}$
Farnesol	✓$_{R,G}$	
Gamma-Gurjunene	✓	
Gamma-Muurolene		✓$_{G}$
Gamma-Terpinene		✓
Geranylgeraniol	✓	
Globulol	✓$_{G}$	✓$_{G}$
Isoaromadendrene epoxide		✓

Juniper camphor (Eudesm-7(11)-en-4-ol)	✓	
Lanosterol	✓	✓
Ledene	✓	
Ledol	✓	✓
Limonene		✓G
Methyl palustrate		✓G
Myrcene		✓
Myrtenol	✓	✓
Neral (2,6-Octadienal, 3,7-dimethyl-)	✓	✓
Nerolidol	✓G	✓G
O-cymene		✓
Oplopanone	✓	
Perilla Alcohol (Para-mentha-1,8-dien-7-ol)	✓	✓
Perillene	✓	
Pinanediol		✓
Pinocarveol	✓	✓
Pinocarvone (Alpha-Pinocarvone)	✓	✓
Sabinene (Sabinene, (1R)-isomer)	✓	✓

Spathulenol	√G	√G
Squalene	√	
Thuja-2,4(10)-diene		√
Verbenone (D-Verbenone)	√R,G	√R,G
**Aldehydes**		
2-isopropenyl-5-methylhex-4-enal	√	
8-hexadecenal, 14-methyl-, (z)-		√
Alpha-Campholenal (Campholenic aldehyde)	√	√
Germacrene D	√G	√G
Phellandral		√
Urs-12-en-28-al	√	
**Ketones**		
Guaiacylacetone	√	
Megastigmatrienone	√	√
**Ethers**		
Verbenyl ethyl ether		√
**Esters and Fatty acids**		
Benzyl benzoate	√R,G	

Dodecanoic acid	✓	
Heneicosane		✓R,G
Hexadecanoic acid	✓	
Nonanoic acid	✓	
Ethyl oleate	✓	✓
Oleic acid	✓	
Phthalic acid (1,2-Benzenedicarboxylic acid)		✓
**Alcohols**		
3,7-nonadien-2-ol, 4,8-dimethyl-	✓	✓
9,19-cyclolanostan-3-ol, 24-methylene-, (3.beta.)-	✓	
Humulane-1,6-dien-3-ol	✓	
Selina-6-en-4-ol	✓	
Trans-3(10)-caren-2-ol	✓	
**Others**		
2-octene, 2-methyl-6-methylene-	✓	✓
Biphenylene, 1,2,3,6,7,8,8a,8b-octahydro-4,5-dimethyl-		✓
Decane		✓
Oxirane, [(dodecycloxy)methyl]-	✓	

N-butylpyrrole	✓

Note: ✓ = detected; Subscripts R and G indicate compounds also identified in red and green propolis references, respectively.

Literature data sources:

64. Alencar, S.M.; Oldoni, T.L.C.; Castro, M.L.; Cabral, I.S.R.; Costa-Neto, C.M.; Cury, J.A.; Rosalen, P.L.; Ikegaki, M. Chemical Composition and Biological Activity of a New Type of Brazilian Propolis: Red Propolis. *Journal of Ethnopharmacology* 2007, *113*, 278–283, doi:10.1016/j.jep.2007.06.005.[TP1]

65. Nunes, C.A.; Guerreiro, M.C. Characterization of Brazilian Green Propolis throughout the Seasons by Headspace GC/MS and ESI-MS. *J Sci Food Agric* 2012, *92*, 433–438, doi:10.1002/jsfa.4596[TP2] .

[TP1]Referencia da própolis vermelha: tabela GC-MS suplementar S1

[TP2]Referencia da própolis verde: tabela GC-MS suplementar S1
