# Supplementary material for: South Brazilian Amber Propolis: Chemical Profile, and Antioxidant, Antimicrobial and Antileukemic Activities
Source: Molecules. 2026 Sep 8;31(18):3143. doi: 10.3390/molecules31183143 (PMC13609837; doi:10.3390/molecules31183143)
Supplement: Supplementary file 1 [file molecules-31-03143-s001.zip › Table S4.pdf]

Table S4: Putative annotation of chemical constituents in South Brazilian amber propolis by LC-MS/MS (positive ionization mode), including chemical formulas, adducts, SMILES notations, SIRIUS/Tanimoto similarity scores, and compound classifications

Candidate Formula	m/z	Adduct	SIRIUS score	Chemical Structure Annotation	TANIMOTO score	Class of compounds	Putative annotation
**C27H30O15**	595.17	[M+H]+	98%	C1=CC(=CC=C1C2=CC(=O)C3=C(C(=C(C(=C3O2)C4C(C(C(C(O4)CO)O)O)O)O)C5C(C(C(C(O5)CO)O)O)O)O)O	100%	Flavonoid C-glycosides	Vicenin-2
**C26H28O14**	565.16	[M+H]+	97.80%	C1C(C(C(C(O1)C2=C(C(=C3C(=C2O)C(=O)C=C(O3)C4=CC=C(C=C4)O)C5C(C(C(C(O5)CO)O)O)O)O)O)O)O	100%	Flavonoid C-glycosides	Vicenin-1
**C27H30O14**	579.17	[M+H]+	99.53%	CC1C(C(C(C(O1)C2=C(C(=C3C(=C2O)C(=O)C=C(O3)C4=CC=C(C=C4)O)C5C(C(C(C(O5)CO)O)O)O)O)O)O)O	99.038%	Flavonoid C-glycosides	Isoviolanthin
**C16H24O5**	297.17	[M+H]+	100%	CC(C1=CC(=O)C2(CCCC(C2C1O)(C)O)C)C(=O)OC	73.62%	Terpenoids	1-acetoxi-labdan-14-al-15-ol

**C36H36O17**	741.2	[M+H]+	97.62%	COC1=C(O)C=CC(=C1)C=CC(=O)OC2C(OC(CO)C(O)C2O)C3=C(O)C=C(O)C4=C3OC(=CC4=O)C5=CC=C(O)C=5	84.32%	Flavonoid C-glycosides	Isoschaftoside 2’’-ferulate
**C20H22O6**	376.18	[M+H]+	99.91%	COC1=C(C=C(C=C1)CC2C(COC2=O)CC3=CC(=C(C=C3)O)OC)O	100%	Dibenzylbutyrolactone lignans	Matairesinol
**C16H14O5**	287.09	[M+H]+	99.97%	COC1=CC(=C2C(=C1)OC(C(C2=O)O)C3=CC=CC=C3)O	79.37%	Flavonoid	Alpinone
**C20H32O5**	353.23	[M+H]+	100%	CC(C)C1(CCC2(C(=C1)C(CC3C2(CCCC3(C)C(=O)O)C)O)O)O	77.27%	Diterpenoids	Abietane-type diterpenoids
**C15H20O4**	265.14	[M+H]+	100%	CC1C2CCC(=CCCC(=CC2OC1=O)C)C(=O)O	74.78%	Sesquiterpenoids	13-epi-manoyl-oxo-labd-8(20)-en-15-oico acid
**C21H24O6**	390.19	[M+H]+	97.52%	COC1=C(C=C(C=C1)CC2COC(=O)C2CC3=CC(=C(C=C3)O)OC)OC	100%	Dibenzylbutyrolactone lignans	Arctigenin-like lignan

**C20H30O4**	335.22	[M+H]+	100%	CC1(CCCC2(C1CCC34C2(CC(CC3)C(=C)C4O)O)C)C(=O)O	84.16%	Diterpenoids	15-hydroxylabdanoico acid
**C15H22O3**	251.16	[M+H]+	100%	CC1C(O)CCC2(C)C1=CC(C(C)C)=C(C2)C(=O)O	80.48%	Sesquiterpenoids	3-hydroxyabiet-8(14)-enoico acid
**C16H26O3**	284.22	[M+H]+	100%	CC1=CC2CC(CCC2C1(C)C(=O)O)C(C)(C)O	71.22%	Sesquiterpenoids	Hydroxyeudesmanonaico acid
**C20H28O5**	349.2	[M+H]+	100%	CC12CCCC(C1CCC(=C)C2CCC3=CC(OC3=O)O)(C)C(=O)O	77%	Diterpenoids	Epsillon-hydroxypinusolidic acid
**C14H22O3**	239.16	[M+H]+	100%	CC(=O)C1=CC(C2(CCCC(C2C1)(C)O)C)O	72.43%	Terpenoids	Trimethyl-hexahydronaphthalene derivative I
**C21H28O7**	415.18	[M+H]+	99.99%	CCC(C)C(=O)OC1C2C(C=C(CCC=C(C1OC)C=O)CO)OC(=O)C2=C	73.60%	Sesquiterpenoids	Germacranolide derivative
**C20H32O4**	354.26	[M+Na]+	100%	CC(=CCO)C(CC1C(=C)CCC2C1(CCCC2(C)C(=O)O)C)O	86%	Diterpenoids	Labdane dicarboxilic acid

**C20H28O2**	301.22	[M+H]+	100%	CC12CCCC3(C1CCC4C2CC=C5C4CCC5(C)C(=O)O)C	86.91%	Diterpenoids	Grandiflorenic acid
**C20H30O3**	319.23	[M+H]+	100%	CC(C)C1=C(O)CC2C1(C)CCC3C2(C)CCCC3(C)C(=O)O	81.60%	Diterpenoids	Abietane-type diterpenoids
**C20H34O5**	354.24	[M+Na]+	100%	CC(C)C1CCC2(C)C1CCC3C2(CCCC3(C)C(=O)O)O(O)O	79.09%	Diterpenoids	Abietane-type diterpenoids
**C20H32O2**	305.25	[M+H]+	100%	CCC1(CCC2C(=C1)CCC3C2(CCCC3(C)C(=O)O)C)C	90%	Diterpenoids	Pimar-8(14)-en-18-oic acid
**C21H22O6**	388.18	[M+H]+	99.98%	COC1=C(C=C(C=C1)C2C3COC(C3CO2)C4=CC(=C(C=C4)OC)OC5O)OC	100%	Furofuranoid lignans	Bursehernin
**C23H24O8**	429.15	[M+H]+	95.56%	COC1=CC(=CC(=C1OC)OC)C2C3C(CC4=C(C5=C(C=C24)OCO5)OC)COC3=O	85.58%	Arylnaphthalene and aryltetralin lignans	Isopicropodophyllin
**C15H24O2**	237.19	[M+H]+	100%	C1=CCC2C(CCC2(CC1=O)C)C(C)(C)O	77%	Sesquiterpenoids	Hexahydroazulen-5-one derivative

**C19H26O2**	287.2	[M+H]+	99.97%	CC(=CCC1C(=C)C=CC2C1(CCC C2C(=O)O)C)C=C	73.36%	Diterpenoids	Abietane-type diterpenoids
**C20H28O4**	332.2	[M+H]+	100%	CC12CCCC(C1CCC(=C)C2CC3C (COC3=O)O)(C)C(=O)O	78%	Diterpenoids	Pinusolidic acid
**C20H16O6**	353.1	[M+H]+	99.99%	CC1=C2C(=CC3=C(O2)C(=O)C(=CO3)C4=CC=C(C=C4)O)OC5= CC=CC=C15	71.60%	Lignans, neolignans and related compounds	Venkatasin derivative
**C15H20O**	271.16	[M+H]+	99.99%	CC1=CC=CC2(C1CC(=C(C)C)C(=O)C2)C	79.05%	Sesquiterpenoids	Eudesman-dien-ona
**C20H26O3**	297.19	[M+H]+	99%	CC(C)C1=C(O)C(O)=C2C(=C1)C 3C(CCC4(C3CC2=O)C)C(C)(C)C (=O)O	83%	Diterpenoids	Rosmanol derivative
**C15H20O5**	281.14	[M+H]+	100%	CC1C2CCC3(C(C2OC1=O)C(C= CC3=O)(CO)O)C	75.25%	Sesquiterpenoids	Hydroxy-guaianolide derivative
**C20H34O4**	339.25	[M+H]+	100%	CC12CCCC(C1CCC(=C)C2CCC(CCO)CO)(C)C(=O)O	86.27%	Diterpenoids	Dihydroxy-oxo-cycloretinol
**C16H32O4**	289.24	[M+H]+	100%	OCC(O)CCCCCCCCCC(=O)O	78.83%	Long chain fatty acid	Hexadecanoic acid, 8,16-dihydroxy
**C20H28O4**	333.21	[M+H]+	100%	CC(C)C1=C(C(=C2C(=C1)CCC3 C2(CCCC3(C)C)C(=O)O)O)O	89.47%	Diterpenoids	Abietane-type diterpenoids

**C20H30O2**	303.23	[M+H]+	100%	CC1(C)CCCC2(C1CCC(=C)C2CC=C(C)C(=O)O)C	96.73%	Diterpenoids	Communic acid
**C20H32O3**	321.24	[M+H]+	100%	CC(=CCO)CCC1C(=C)CCC2C1(CCCC2(C)C(=O)O)C	91.58%	Diterpenoids	Isocupressic acid
**C20H34O2**	307.26	[M+H]+	100%	CC1(C)CCCC2(C)C1CCC(C2)C(C)CCCC(C)C(=O)O	83.15%	Diterpenoids	Abietane-type diterpenoids
**C16H26O2**	251.2	[M+H]+	99.99%	CC1(CCCC2(C1CCC3(C2CC(=O)O3)C)C)C	97.53%	Sesquiterpenoids	Sclareolide
**C20H32O4**	337.24	[M+H]+	100%	CC(CCC1C(=C)CCC2C1(CCCC2(C)C(=O)O)C)CC(=O)O	88.67%	Diterpenoids	Pinifolic Acid
**C20H28O**	285.22	[M+H]+	100%	CC(C)C1=C(C=C2C=CC3C(CCCC3(C2=C1)C)(C)C)O	88.37%	Diterpenoids	Ferruginol
**C16H30O2**	255.23	[M+H]+	100%	CCCCC/C=C\CCCCCCCC(=O)O	99%	Long chain fatty acid	Zoomarate
**C20H34O**	291.27	[M+H]+	100%	CC(=CCCC(=CCO)C)CCC1C(=C)CCCC1(C)C	74.26%	Diterpenoids	Trixagol

**C30H46O3**	455.35	[M+H]+	100%	CC1CCC2(C(C1C)C3=CCC4C5(CCC(C(C5CCC4(C3CC2)C)(C)C)O)C)C(=O)O	86.99%	Triterpenoids	Ursolic acid
**C18H32O2**	281.25	[M+H]+	100%	CCCCCCC=CCC=CCCCCCCCC(=O)O	93.70%	Linoleic acid and derivatives	Linolate
**C30H46O4**	471.25	[M+Na]+	99.98%	CC1(C)CCC2(C1C3=C(CC2)C4(C(CC3)C5(C(CC(C(C5CC4)O)(C)C)=O)C)C)C(=O)O	85.53%	Triterpenoids	Hydroxy-oxo-oleanoic acid isomer
**C20H38O3**	327.29	[M+H]+	100%	CCCCCCC(CC=CCCCCCCCCCC(=O)O)O	82.65%	Fatty alcohols	Lesquerolate
**C20H32**	273.26	[M+H]+	100%	CC1(CCCC2(C1CCC(=C)C2CCC(=C)C=C)C)C	83%	Diterpenoids	Sclarene
**C18H35NO**	282.28	[M+H]+	100%	CCCCCCCCC=CCCCCCCCC(=O)N	100%	Lipids	Oleamide
**C31H50O4**	487.34	[M+Na]+	99%	CC1(CCC2(C(CC3(C(=C2C1)CCC4C3(CCC5C4(CCC(C5(C)C)O)C)C)O)C(=O)OC)C)C	89.15%	Triterpenoids	Regelindiol B

**C20H42O4**	347.32	[M+Na]+	100%	CCCCCCCCCCCCCCCCC(C(C(CO)O)O)O	83.18%	Monoacylglycerols	Icosane-1,2,3,4-tetrol
**C29H46O2**	427.26	[M+H]+	100%	CCC(C)C(C)CC(O)C(C)C1CCC2C1(CC=C3C2CCC4=CC(=O)CCC43C)C	73.14%	Triterpenoids	Haloxysterol C
**C30H48O3**	457.37	[M+H]+	100%	CC(=CCCC(C1CCC2(C1(CCC3=C2CCC4C3(CCC(C4(C)C)O)C)C)C)C(=O)O)C	77.04%	Triterpenoids	Trametenolic acid
**C30H50O2**	443.39	[M+H]+	99.99%	CC1CCC2(CCC3(C(=CCC4C3(CCC5C4(CCC(C5(C)C)O)C)C)C2C1C)C)CO	92.95%	Triterpenoids	Uvaol
**C30H46O2**	439.36	[M+H]+	99.79%	CC1C2C3=CC(=O)C4C5(CCC(C(C5CCC4(C3(CCC2(CC=C1C)C)C)C)©C)O)C	88.18%	Triterpenoids	Ursolic acid lactone
**C30H48O2**	441.37	[M+H]+	100%	CC1(C)C2CCC3(C)C(C2(C)CC1)C(=O)C=C4C5C(C)(C)CCC5(C)CCC43C	92.34%	Triterpenoids	11-oxo-beta-amyrin

**C20H39NO**	310.31	[M+H]+	100%	CCCCCCCCC=CCCCCCCCC(=O)NCC	74.57%	Lipids	Oleoyl Ethyl Amide
**C21H40O2**	325.31	[M+H]+	100%	CCCCCCC=CCCCCCCCCCCC(=O)OC	95.45%	Fatty acid methyl esters	Methyl Icos-13-enoate
**C31H48O4**	485.36	[M+Na]+	98.21%	CC1(C)CCC2(C)C(CCC3(C)C2C(=O)C=C4C5C(C)(C)CC(O)CC5(C)CCC43C)C1C(=O)OC	75.89%	Triterpenoids	Methyl β-glycyrrhetinate
**C20H36O2**	309.28	[M+H]+	100%	CC(C)CC1C(=C)CCC2C1(CCCC2(C)O)C(C)(C)O	77%	Diterpenoids	Sclareol
**C30H46O**	422.36	[M+H]+	99.96%	CC(=C)C1CCC2(C1C3=CCC4C5(CCC(=O)C(C5CCC4(C3(CC2)C)C)(C)C)C)C	76.08%	Triterpenoids	Lupadienone
**C15H24**	205.2	[M+H]+	100%	CC(=C)C12CC(C1CCC2C(=C)C)C	78.43%	Sesquiterpenoids	Bicyclo[3.2.0]heptane derivative
**C23H46O3**	371.25	[M+Na]+	100%	CCCCCCCCCCCCCCCCCCC(=O)OCC(CO)O	78.57%	Monoacylglycerols	1-Monoeicosanoin
**C30H48O**	425.38	[M+H]+	100%	CC1(C)CCC2(C)C1CC=C3C2(C)CCC4(C)C3CCC5(C)C4CCC(=O)C5(C)C	89.65%	Triterpenoids	β-amirona

**C31H50O4**	487.38	[M+Na]+	100%	CC1(CCC2(C(C1)C3=CCC4C5(CCC(C(C5CCC4(C3(CC2O)C)C)C)O)C)C(=O)OC)C	87.41%	Triterpenoids	Methyl echinocystate
**C21H44O3**	345.34	[M+Na]+	100%	CCCCCCCCCCCCCCCCCCOCC(CO)O	98.40%	Monoacylglycerols	Batilol
**C24H48O3**	349.35	[M+H]+	100%	CCCCCCCCCCCCCCCCCCCCCC(CC(=O)O)O	85.15%	Fatty acids	3-hydroxylignoceric acid
**C24H46O2**	367.36	[M+H]+	100%	CCCCCCCCC=CCCCCCCCCCCCCCC(=O)O	100%	Long chain fatty acids	Nervonate
**C20H38O2**	311.29	[M+H]+	100%	CC(CCC1C(CCC2C1(CCCC2(C)C)C)(C)O)CCO	99.37%	Diterpenoids	Larixol derivative
**C18H36O2**	285.28	[M+H]+	100%	CCCCCCCCCCCCCCCC(=O)OCC	98.50%	Fatty acid esters	Ethyl cetylate
**C32H62O5**	527.47	[M+Na]+	100%	CCCCCCCCCCCCCCCCC(=O)OCC(COC(=O)CCCCCCCCCCC)O	86.17%	Diacylglycerols	1,2-Dipalmitoyl-sn-glycerol
**C31H48O2**	453.37	[M+H]+	100%	CC(C)CCCC(C)C1CCC2(C1CCC34C2CC(C)C5C3(C4)CCC(C)C5(C)C)O	71.96%	Triterpenoids	Cimigenol derivatives

**C31H50O2**	455.39	[M+H]+	100%	CC(=C)C1CCC2(C1C3CCC4C5(CCCC(C5CCC4(C3CC2)C)(C)C)C)C=O	86.12%	Triterpenoids	Betulinaldehyde
**C15H22O2**	235.17	[M+H]+	100%	CC1CC(C=C(C12CCC(C2)C(=C)C)C=O)O	76%	Sesquiterpenoids	Ilicisic acid
**C29H46O**	411.36	[M+H]+	99.99%	CC1(C)C2CCC3(C)C(CCC4C3(CC=C5C4CCC(C)(C)C5=O)C)C2(C)C1	86.44%	Triterpenoids	Ursanol
**C32H52O2**	469.4	[M+H]+	100%	CC(=O)OC1CCC2(C(C1(C)C)CCC3(C2CC=C4C3(CCC5(C4CC(CC5)(C)C)C)C)C)C	85%	Triterpenoids	Methyl oleanolate
**C32H52O2**	469.4	[M+H]+	100%	CC1CCC2(CCC3(C(=CCC4C3(CCC5C4(CCC(C5(C)C)CC(=O)O)C)C)C2C1C)C)C	70%	Triterpenoids	Oleanolic acid
**C13H22O**	195.17	[M+H]+	100%	CC1=CCCC(C1CCC(=O)C)C	92.68%	Terpenoids	Dihydro-alpha-ionone
**C20H30O2**	303.23	[M+H]+	100%	CC1(C)CCCC2(C)C1CCC3(C)C2CCC(=C)C3C(=O)O	96.18%	Diterpenoids	Isopimaric acid

**C36H62O4**	559.47	[M+Na]+	99.65%	CCCCCCCCCCCCCCCCCCCCCCCOC(=O)C=CC1=CC=C(O)C=C1	70.61%	Terpenoids	Phenolic ester terpenoid
**C17H12O8**	345.06	[M+H]+	100%	CC1=C(C=C2C(=C1O)C(=O)OC3=C(C=C(C4=C3OC2=O)C=O)C(=O)O)C	82.77%	Salicylic acid and derivatives	Depsidone derivative
**C16H10O8**	331.05	[M+H]+	100%	CC1=C(C(=O)O)C2=C(C=C1O)C(=O)C3=C(C2=O)C=C(O)C(=C3O)O	70.42%	Anthraquinone/ Benzoic acids	Anthraquinone derivatives
