# Supplementary material for: South Brazilian Amber Propolis: Chemical Profile, and Antioxidant, Antimicrobial and Antileukemic Activities
Source: Molecules. 2026 Sep 8;31(18):3143. doi: 10.3390/molecules31183143 (PMC13609837; doi:10.3390/molecules31183143)
Supplement: Supplementary file 1 [file molecules-31-03143-s001.zip › Table S5.pdf]

Table S5: Putative annotation of chemical constituents in South Brazilian amber propolis by LC-MS/MS (negative ionization mode), including chemical formulas, adducts, SMILES notations, SIRIUS/Tanimoto similarity scores, and compound classifications

Candidate Formula	m/z	Adduct	SIRIUS score	Chemical Structure Annotation	TANIMOTO score	Class of compounds	Putative annotation
**C20H40O3**	327.29	[M−H]−	100%	CCCCCCCCCCC(CCCCCCCCC(=O)O)O	96%	Long chain fatty acids	Hydroxy- eicosanoic acid
**C18H32O2**	279.23	[M−H]−	100%	CCCCCCCCC#CCCCCCCC(=O)O	95.49%	Long chain fatty acids	8-Octadecynoic acid
**C17H12O8**	343.05	[M−H]−	100%	CC1=CC(=C(C2=C1C(=O)OC3=C(O2)C(=CC(=C3C)O)C(=O)O)C=O)O	91.69%	Carboxylic acids and derivates	Dihydroxy-dimethyl-oxobenzodioxepine acid
**C16H32O4**	287.22	[M−H]−	100%	C(CCCCO)CCCC(CCCCCC(=O)O)O	89.17%	Hidroxy acids and derivates	Dihydroxyhexadecanoic acid
**C18H32O3**	295.23	[M−H]−	100%	CCCCCC=CC=CC(CCCCCCC(=O)O)O	86.62%	Fatty acyls	Coriolic acid

**C20H32O3**	319.23	[M−H]−	100%	CCCC(CC=CCC=CCC=CCC=CCCCC(=O)O)O	82.78%	Diterpenoids	17-hydroxyeicosatetraenate
**C15H8O6**	343.05	[M−H]−	100%	C1=CC2=C(C(=C1)O)C(=O)C3=C(C2=O)C=C(C=C3O)C(=O)O	81.35%	Isobenzofuranones	Monorhein
**C30H60O2**	451.45	[M−H]−	100%	CCCCCCCCCCCCCCCCCCCCCCCCCCCCCC(=O)O	80.30%	Long chain fatty acids	Melissic acid
**C20H36O4**	321.24	[M−H]−	100%	CC1CCC2(C(C1(C)CCC(C)CC(=O)O)CCC(C2(C)O)O)C	73%	Diterpenoids	Hydroxylated abietic acid
**C16H10O8**	329.03	[M−H]−	100%	C1=CC(=CC(=C1)C(=O)OC2=CC(=C(C=C2)C(=O)O)C(=O)O)C(=O)O	72.88%	Biphenyls and derivates	Biphenyltricarboxylic acid
**C18H26O3**	289.18	[M−H]−	100%	CCC=CC=C1C(C=CC1=O)CCCCCCCC(=O)O	70.65%	Phenolic acid derivative	Ferulic acid derivative
**C18H34O2**	281.25	[M−H]−	100%	CCCCCCCCC=CCCCCCCCC(=O)O	100%	Long chain fatty acids	Oleic acid

**C16H32O3**	271.23	[M−H]−	100%	C(CCCCCCCC(=O)O)CCCCCCCO	100%	Medium-chain hydroxy acids	Hydroxy-palmitic acid
**C18H36O4**	315.25		99.99%	CCCC(CCCCC(CCCCCCCCC(=O)O)O	82.70%	Long chain fatty acids	12-Hydroxydecanoic acid
**C36H62O4**	557.46	[M−H]−	99.99%	CCCCCC=C(CCCCCCCCC=CCC=CCCCCC(=O)O)CC=CCCCCCCCC(=O)O	82.14%	Fatty acyls	Octatriaconta-trienedioic acid
**C20H28O4**	331.19	[M−H]−	99.99%	CC12CCCC(C1CC=C3C2CC(=O)C(=C3)C(C)(C)O)(C)C(=O	80.57%	Prenol lipids	Dihydroxy-furan-ethyl derivative
**C40H78O7**	669.57	[M−H]−	99.99%	CCCCCCCCCCCCCCCCCCCCCCCCCCC(=O)OCC(COC(=O)CCCCCCC)O	80.22%	Fatty acyls	Glyceryl ester derivative
**C20H30O3**	317.21	[M−H]−	99.99%	CC12CCCC(C1CCC3=CC(C(CC23)O)(C)C=C)(C)C(=O)O	76.64%	Prenol lipids	Labdane-type diterpenoid

**C22H36O4**	363.25	[M−H]−	99.99%	CC12CCC(CC1CCC(C2)C3CCCC(=CCO)C3(C)CC(=O)O)O	72.50%	Diterpenoids	Acetyloxy-hexahydronaphthalene acid
**C21H30O3**	329.21	[M−H]−	99.99%	CC(C)C1=C(C=C2C(=C1OC)CCC3C2(CCC(=O)C3(C)C)O)O	72.48%	Prenol lipids	O-methylpisiferic acid
**C17H14O6**	313.07	[M−H]−	99.99%	CCOCC1=C2C(=CC(=C1)O)C(=O)C3=CC(=CC(=C3C2=O)O)O	70.78%	Anthraquinones	Dihydroxy-tricarboxy anthraquinone
**C20H38O2**	309.28	[M−H]−	99.98%	CCCCCCCCCC=CCCCCCCCCC(=O)O	99.24%	Long chain fatty acids	Gondoic acid
**C15H20O4**	263.13	[M−H]−	99.98%	CC1C(=O)OCC2C13CC(CC3C=C2C(=O)O)(C)C	73.66%	Prenol lipids	Pentalenolactone D derivative
**C20H28O**	283.21	[M−H]−	99.97%	CC(=C)C1=C(C=CC2=C1CCC3C2(CCCC3(C)C)C)O	89.95%	Diterpenoids	Cedrol derivative
**C20H30O4**	333.21	[M−H]−	99.97%	CC1(CCC2C(=C1)CCC3C2(CCCC3(C)C(=O)O)C)CC(=O)O	80.18%	Prenol lipids	Octahydrophenanthrene-1-carboxylic acid

**C20H32O4**	335.22	[M−H]−	99.97%	CC12CCCC(C1CCC34C2CC(C(C3)(CC4)CO)O)(C)C(=O)O	73.95%	Corticoids	Hydrocortisone acid derivative
**C19H32O3**	307.23	[M−H]−	99.96%	CC(CCC1C(=C)CCC2C1(CCCC2(C)C(=O)O)C)CO	72.85%	Diterpenoids	15-Hydroxy-13,14-dihydroabietic acid
**C20H28O2**	299.2	[M−H]−	99.94%	CC(C)C1=C(C=C2C(=C1)CCC3C2(CCCC3(C)C)C=O)O	89.68%	Diterpenoids	13-Isopropylpodocarpen-12-ol-20-al
**C20H40O2**	311.29	[M−H]−	99.91%	CCCCCCCCCCCCCCCCCCCC(=O)O	99%	Long chain fatty acids	Arachidic acid
**C14H24O4**	239.17	[M−H]−	99.90%	C(CCCCCC(=O)O)CCCCC#CCO	81%	Fatty acyls	13-hydroxy-10-tetradecynoic acid
**C18H36O2**	283.26	[M−H]−	99.89%	CCCCCCCCCCCCCCCCCC(=O)O	100%	Fatty acyls	Stearic acid derivative

**C15H24O3**	251.17	[M−H]−	99.88%	CC(C)CCCC(C)(C1=CC=C(C=C1)CO)O)O	70.76%	Phenylpropanes	Phenylpropanoid derivative
**C16H12O5**	283.06	[M−H]−	99.87%	COC1=CC(=C2C(=C1)OC=C(C2=O)C3=CC=C(C=C3)O)O	92%	Flavonoids	Prunetin
**C22H36O2**	391.29	[M−H]−	99.87%	CCCCCCCCC(CCCCCCC(=O)O)C1=CC=CC=C1	74.46%	Benzenoids	Phenyl-substituted fatty acid
**C30H48O5**	487.34	[M−H]−	99.85%	CC1CCC2(CCC3(C(=CCC4C3(CCC5C4(CCC(C5(C)CO)O)C)C)C2C1(C)O)C)C(=O)O	85.66%	Triterpenoids	Rotundic acid
**C31H52O3**	471.38	[M−H]−	99.78%	CC1CCC(C(C1CCC2(C(CCC3C2(CCC4C3(CC(CC4)C(=C)C)C)C(=O)O)C)C)C)O	71.31%	Prenol lipids	3β-hydroxy-urs-12-en-28-oic acid derivative
**C16H24O4**	279.16	[M−H]−	99.74%	COC1=CC=CC(=C1C(=O)O)CCCCCCCCO	84.95%	Benzoic acids	4-Methoxybenzoic acid derivative

**C32H62O4**	509.46	[M−H]−	99.74%	CCCCCCCCCC(CCCCCCC(=O)O)C(CCCCCCCCCCCC)OC(=O)C	74.32%	Fatty acids esters	Hexadecanoic Diester
**C20H26O2**	297.19	[M−H]−	99.57%	CCCCCC1=CC(=C2C3C=C(CCC3C(=C)OC2=C1)C)O	84.42%	Benzopyrans	Benzopyran derivative
**C21H34O2**	317.25	[M−H]−	99.53%	CCCCCCC=CCCCCC1=C(C(=CC=C1)O)O	84.70%	Phenolic lipid	Urushiol II
**C24H40O5**	407.28	[M−H]−	99.53%	CC12CCC3C(C1CCC2C(C)(CCC(=O)O)O)CC(C4C3(CCC(C4)O)C)O	70%	Hydroxy bile acids, alcohols and derivates	Steroid derivative
**C30H48O4**	471.35	[M−H]−	99.37%	CC1CCC2(CCC3(C(=CCC4C3(CC(C5C4(CCC(C5(C)C)O)C)O)C)C2C1(C)C)C)C(=O)O	90.87%	Triterpenoids	Tetradecahydropicene-carboxylic acid
**C18H16O7**	343.08	[M−H]−	99.17%	COC1=CC2=C(C(=C1)OC)C(=O)C=C(O2)C3=CC(=C(C(=C3)O)OC)O	89.47%	Flavonoids	Lethedocin derivative

**C36H68O4**	563.5	[M−H]−	99%	CCCCCCCCCC(CCCCCCC C(=O)O)OC(=O)CCCCCC CC=CCCCCCCCC	75.77%	Fatty acid esters	Stearic acid diester
**C21H28O4**	343.19	[M−H]−	99%	CC(CC1=CC(=C(C=C1)O C)O)C(C)CC2=CC(=C(C= C2)OC)OC	70%	Phenolic derivative	Dimethoxyphenyl derivative
**C20H26O3**	313.18	[M−H]−	98.99%	CC(C)C1=CC2=C(C=C1)C 3(CCCC(C3CC2=O)(C)C(=O)O)C	84.30%	Prenol lipids	7-oxocallitrisic acid
**C34H66O4**	537.49	[M−H]−	98.74%	CCCCCCCCCCCCCCCC(= O)OC(=O)CCCCCCCCCC(CCCCCCC)O	81.43%	Fatty acids esters	Palmitic acid Diester
**C17H16O4**	283.1	[M−H]−	98.74%	C1=CC=C(C=C1)CCOC(= O)C=CC2=CC(=C(C=C2) O)O	100%	Phenolic acid derivative	Caffeic acid phenethyl ester (CAPE)
**C20H28O6**	363.18	[M−H]−	98.71%	CC1(CC23CC1CCC2C45 CCC(C(C4C3C(=O)O)(C(=O)O5)C)O)OC	71.14%	Dicarboxylic acids and derivates	2,3-Dicarboxybenzofuran acid
**C22H42O2**	337.31	[M−H]−	98.55%	CC(=C)CCCCCCCCCCCC CCCCCC(=O)O	81.30%		Cetoleate

						Long chain fatty acids	
**C14H22O4**	253.14	[M−H]−	98%	CC(=O)CCCCCCC(C1COC(=O)C1=C)O	76.63%	Carboxylic acids and derivates	2,11-dimethylidenedodecanedioic acid
**C23H32O6**	449.22	[M−H]−	97.38%	CC1CC2C3CCC(C3(CC(C2C4(C1=CC(=O)CC4)C)O)C)(C(=O)CC(=O)O)O	71.87%	Hydroxy bile acids, alcohols and derivates	7-Hydroxycolic acid derivative
**C15H22O4**	265.14	[M−H]−	96.83%	CC1(CCCC2(C1C(C=C3C2(C(=O)OC3)O)O)C)C	74.15%	Prenol lipids	Pereniporin B
**C30H46O3**	453.34	[M−H]−	94.98%	CC1(CCC2(CCC3(C(C2=C1)CCC4C3(CCC5C4(CCC(=O)C5(C)C)C)C)C)C(=O)O)C	84.54%	Prenol lipids	Moronic acid
**C27H30O15**	593.15	[M−H]−	94%	C1=CC(=CC=C1C2=CC(=O)C3=C(C(=C(C(=C3O2)C4C(C(C(C(O4)CO)O)O)O)O)C5C(C(C(C(O5)CO)O)O)O)O)O	100%	Flavonoid C-glycoside	Vicenin-2

**C25H40O4**	449.29	[M−H]−	93.61%	CC(CC(C)(C)C(=O)O)C1CCC2C1(CCC3C2C(=O)CC4C3CCC(C4)O)C	71.25%	Hydroxy bile acids, alcohols and derivates	3-Hydroxy-7,12-dioxocolic acid
**C15H24O4**	267.16	[M−H]−	92.02%	CC1CCC(CC1=O)(C(C)CC(=O)O)C(=O)C(C)C	85.46%	Prenol lipids	Acoric acid
**C38H68O4**	587.5	[M−H]−	91.45%	CCCCCCC1C=CC(C(C1CCCCCC)C=CCCCCCCCC(=O)O)CCCCCCCCCC(=O)O	70.37%	Fatty acyls	Carboxynon-cyclohexen-decanoic acid
**C34H62O4**	533.46	[M−H]−	90.57%	CCCCCCC=CCCCCCCC(CCCCCCCCC=CCCCCCCCC(=O)O)C(=O)O	76.41%	Fatty acyls	Docosadienyl-octadecenedioic acid
**C20H34O5**	353.23	[M−H]−	88.45%	CCCCCC(C=CC1C(C(CC1=O)O)CCCCCCC(=O)O)O	77.37%	Prostaglandins and related compounds	Prostaglandin D1 derivative
**C16H12O6**	299.00	[M−H]−	88.43%	COC1=C(OC2=CC(=CC(=C2C1=O)O)O)C3=CC=C(C=C3)O	96.15%	Flavonoids	Isokaempferide

**C18H28O3**	291.2	[M−H]−	78%	CCCC=CC=CC=CC(=O)C CCCCCCC(=O)O	77%	Medium-chain fatty acids	11-Oxo-octadeca-4,6,8-trienoic acid
**C19H16O8**	371.08	[M−H]−	70.81%	CC1=CC(=C(C2=C1C(=O) OC3=C(O2)C(=C(C(=C3C)O)C(=O)OC)C)C=O)O	75.50%	Naphtalenes	Granulatin derivative
**C15H10O5**	269.05	[M−H]−	100%	C1=CC(=CC=C1C2=CC(= O)C3=C(C=C(C=C3O2)O)O)O	100%	Flavonoids	Apigenin

Table S5: Putative annotation of chemical constituents in South Brazilian amber propolis by LC-MS/MS (negative ionization mode), including chemical formulas, adducts, SMILES notations, SIRIUS/Tanimoto similarity scores, and compound classifications
